# RFLNA mitigates heat stress-impaired chondrocyte proliferation and vertebral development through cytoskeletal regulation in pigs

**DOI:** 10.1186/s40104-026-01387-x

**Published:** 2026-04-15

**Authors:** Xiaoyang Yang, Yuxuan Xie, Yabiao Luo, Yubei Wang, Lixian Yang, Longmiao Zhang, Pengxiang Xue, Chengwan Zha, Meiying Fang

**Affiliations:** 1 State Key Laboratory of Animal Biotech Breeding, National Engineering Laboratory for Animal Breeding, MOA Key Laboratory of Animal Genetics and Breeding, College of Animal Science and Technology, Frontiers Science Center for Molecular Design Breeding, Beijing Key Laboratory for Animal Genetic Improvement, China Agricultural University, Beijing, 100193 China; 2 Sanya Institute of China Agricultural University, Sanya, 572000 China

**Keywords:** Body length, Chondrocyte development, Heat stress, Pig, RFLNA

## Abstract

**Background:**

Global climate change has brought severe challenges to the livestock industry, among which heat stress (HS) acts as a key factor impairing the growth and development of pigs. It has been established that ambient temperature influences body size traits mainly by directly affecting skeletal development, yet the molecular mechanisms by which HS inhibits this process remain largely unclear. The cytoskeleton is critical for mediating cellular morphological adaptation to environmental stimuli, and HS can disturb cellular development by destroying cytoskeletal homeostasis. Previous studies have demonstrated that *RFLNA* regulates bone development through the cytoskeleton, but whether it alleviates HS-induced chondrocyte damage by modulating the cytoskeleton has not been reported. Therefore, this study was conducted to explore the effects of HS on the proliferation and differentiation of pig thoracic vertebral growth plate chondrocytes (PTVCs), and to clarify the regulatory mechanism by which the regulatory factor *RFLNA* endows PTVCs with HS resistance.

**Results:**

Transcriptomic profiling of PTVCs cultured under control (37 °C) and HS (41 °C) conditions at multiple differentiation time points revealed that HS suppressed cell proliferation and extracellular matrix synthesis. Differentially expressed genes (DEGs) in the early HS response (6 h and 24 h) were enriched in inflammation and stress-response pathways. In contrast, DEGs from later-phases (48, 96, and 144 h) were linked to cytoskeletal reorganization. Further analysis revealed that *RFLNA* expression was upregulated both by HS and during chondrocyte differentiation. *RFLNA* expression was higher in the thoracic vertebrae tissues from large-sized Yorkshire pigs than in those from small-sized Wuzhishan pigs. Spatial expression analysis indicated that *RFLNA* was predominantly expressed in thoracic and lumbar vertebrae, with subcellular localization to the cytoskeleton. Functional assays demonstrated that *RFLNA* overexpression under HS conditions promoted PTVC proliferation and adhesion while inhibiting migration, thereby mitigating HS-induced growth suppression. Conversely, *RFLNA* knockdown exacerbated the detrimental effects of HS.

**Conclusions:**

Our findings show *RFLNA* might be a critical mediator of thermal adaptation and vertebral development, which links cytoskeletal regulation to temperature-related variations in body size. This work provides a theoretical foundation for strategies aimed at enhancing climate resilience in swine.

**Supplementary Information:**

The online version contains supplementary material available at https://doi.org/10.1186/s40104-026-01387-x.

## Background

With the intensification of global warming, the increased frequency of HS environments disrupts normal bone development, leading to substantial economic losses in livestock production [1]. Pigs have a normal body temperature range of 38–39 °C [2]. Their limited capacity for evaporative heat dissipation due to underdeveloped sweat glands makes them heavily reliant on respiratory cooling and environmental modification for thermoregulation [3]. In poorly ventilated, high-humidity environments, heat stress (HS) can elevate a pig's core temperature above 40 °C and skin temperature beyond 41 °C [4]. Bone tissue, being relatively superficial, is directly exposed to ambient temperature fluctuations [5]. This thermal influence can impair the developmental process directly, bypassing indirect physiological pathways such as endocrine or metabolic signaling [6]. Given the close correlation between animal body size and environmental temperature, and the fact that skeletal development is a key determinant of porcine production performance, identifying strategies to mitigate the adverse effects of HS on growth presents a critical scientific and industrial challenge in animal genetics, breeding, and reproduction.

As the core tissue governing longitudinal bone growth, the epiphyseal growth plate relies on a precise balance of chondrocyte proliferation, differentiation, and apoptosis. HS disrupts this homeostasis, inducing chondrocyte apoptosis and functional dysregulation, which ultimately impairs skeletal development [7]. However, the specific molecular mechanisms involved are not well characterized, and effective regulatory targets to counteract these effects remain elusive. Refilin-A (RFLNA, also known as CFM2 or FAM101A) is a vertebrate-specific filamin-binding protein and a member of the newly identified family of filamin-binding proteins (RFLN), which modulates the actin-filamin interaction during skeletogenesis [8]. Studies in mice have demonstrated that knockout of *Rflna* and *Rflnb* results in severe skeletal abnormalities, including shortened distal appendages, scoliosis, kyphosis, intervertebral disc defects, vertebral body fusion, and delayed longitudinal bone growth [8, 9]. In humans, reduced *RFLNA* expression is associated with adolescent idiopathic scoliosis, underscoring its conserved and essential role in vertebral column development [10]. Despite this evidence, the precise function of *RFLNA* in vertebral tissues, particularly under environmental stress, requires further investigation.

The interaction between environmental temperature and genetic factors significantly influences the development of body conformation traits in pigs. We found that low expression of *RFLNA* in pigs from high-temperature regions is associated with shorter body length traits. We hypothesize that under HS conditions, *RFLNA* functions as a stress-responsive molecule to alleviate HS-induced suppression of chondrocyte proliferation. Investigating this role is crucial for understanding the genetic basis of thermal adaptation and holds significant potential for breeding pigs with enhanced resilience to climate change.

## Methods

### Isolation and culture of primary PTVCs

PTVCs were isolated from the thoracic vertebral growth plates of three neonatal (1–3 days old) Yorkshire pigs at the Beijing Pig Breeding Farm (Beijing, China) to establish an in vitro model system. Under aseptic conditions, surrounding connective tissues were carefully removed from the cartilage using a scalpel and surgical scissors. The tissue fragments were immersed in 75% ethanol for 30 s, rinsed with PBS for 30 s, and this rinse cycle was repeated five times. The cartilage was then minced into ~ 1 mm^3^ fragments and digested for 8 h at 37 °C in DMEM/F12 medium containing 2% fetal bovine serum (FBS), 3% penicillin–streptomycin, 1% amphotericin B, and 0.2% collagenase II, with gentle agitation every 30 min. The resulting cell suspension was filtered through a 40 μm nylon cell strainer and centrifuged at 300 × *g* for 10 min. The collected cells were resuspended and cultured in growth medium (DMEM/F12 supplemented with 10% FBS and 1% penicillin–streptomycin) at 37 °C with 5% CO_2_ for 48 h. The complete medium was replaced according to the cell attachment status, and the basic medium was replaced every 2 d thereafter. Upon reaching confluence, cells were switched to chondrogenic differentiation medium (DMEM/F12 containing 5% FBS, 1% penicillin–streptomycin, and 1% insulin-transferrin-selenium (Gibco, Grand Island, NY, USA)) to induce differentiation. For HS treatment, cells were incubated at 41 °C with 5% CO₂ in a Celstar185 incubator (Radobio, Shanghai, China).

### Cell Counting Kit-8 (CCK-8) assay

PTVCs were seeded in a 96-well plate (Corning, NY, USA) at a density of 3 × 10^3^ cells per well in 100 μL of growth medium (*n* = 6). According to the manufacturer's protocol, the CCK-8 (C0038, Beyotime Biotechnology, Shanghai, China) was used to monitor cell proliferation at 41 °C and 37 °C for 48 h. After adding the CCK-8 reagent and incubating for 1 h, the absorbance was measured at 450 nm using a SpectraMax^®^ i3x Multi-Mode Microplate Reader (Molecular Devices Corporation, Sunnyvale, California, USA).

### 5-Ethynyl‐2′‐deoxyuridine (EdU) assay

PTVCs were inoculated at a density of 2 mL containing 5 × 10^4^ cells per well into a 12-well plate (Corning, NY, USA) and cultured in basic medium (*n* = 4). After culturing at 41 °C for 48 h, according to the manufacturer's protocol, the cells were stained with Alexa Fluor 555 for 2 h using the BeyoClick™ EdU Cell Proliferation Kit (C0075L, Beyotime Biotechnology, Shanghai, China). The cell nuclei were stained blue with DAPI, and the EdU-positive cells were stained red. Images were captured using an Echo Revolve Fluorescent Microscope (Echo Laboratories, San Diego, California, USA).

### Alcian blue staining

A total of 1.5 × 10^5^ cells were resuspended in 10 μL of the basic medium and dropped into a 24-well plate (Corning, NY, USA). Cells were adherent for 2 h at 37 °C before 1 mL of growth medium was added. After 48 h, the medium was replaced with chondrogenic differentiation medium, and cells were cultured at either 37 °C or 41 °C. For staining, cells were washed with PBS, fixed with 4% paraformaldehyde for 10–15 min, and rinsed with ddH_2_O. Cells were immersed in an Alcian acidification solution for 3 min, then stained with Alcian blue solution (G1560, Solarbio, Beijing, China) for 30 min. After a final 5-min wash with water, stained cells were imaged in PBS.

### Transcriptome analysis

PTVCs were cultured in 6-well plates under differentiation conditions at 37 °C or 41 °C and harvested at 6, 24, 48, 96, and 144 h. Each condition and time point included three biological replicates, yielding 30 total samples. The 37 °C group was named as C6 (C6-1, C6-2, C6-3), C24 (C24-1, C24-2, C24-3), C48 (C48-1, C48-2, C48-3), C96 (C96-1, C96-2, C96-3), C144 (C144-1, C144-2, C144-3). The 41 °C group was named as H6 (H6-1, H6-2, H6-3), H24 (H24-1, H24-2, H24-3), H48 (H48-1, H48-2, H48-3), H96 (H96-1, H96-2, H96-3), H144 (H144-1, H144-2, H144-3). The total RNA of cell samples was extracted using the TRNzol method (DP424, TIANGEN, Beijing, China). The quality of the total RNA samples was inspected using NanoDrop, and the integrity of the RNA was detected by Bioanalyzer 2100 (Agilent Technologies, Santa Clara, CA, USA). Strand-specific cDNA libraries were constructed from 200 ng of total RNA per sample using SuperScript™ II Reverse Transcriptase (Invitrogen, Waltham, MA, USA) and then subjected to paired-end sequencing on an Illumina HiSeq Novaseq™ 6000 platform.

Raw sequencing reads were quality-filtered using Fastp (v0.23.1) to remove adapter sequences, reads with > 10% ambiguous bases, and low-quality reads (over 50% of bases with Phred score < 5). Clean reads were aligned to the pig reference genome (*Sus scrofa* 11.1, GCF_000003025.6) using HISAT2. Gene expression levels were quantified with featureCounts and normalized to TPM (transcripts per million). Differential expression analysis was performed using the DESeq2 package in R, with genes identified as differentially expressed (DEGs) at *P* < 0.05 and |log_2_ fold change| ≥ 1. Time-series clustering and visualization were conducted with the ClusterGvis package. Temporal expression profiles were analyzed using maSigPro [11]. Functional enrichment analyses [(Kyoto Encyclopedia of Genes and Genomes (KEGG) and Gene Ontology (GO)] were performed with clusterProfiler [12]. Weighted Gene Co-expression Network Analysis (WGCNA) was used to identify HS-associated hub genes.

Public RNA-seq data from thoracic vertebra cartilage tissues of 1-month-old and 4-month-old Wuzhishan and Yorkshire pigs were obtained from PRJNA949733 [13]. Data from northern and southern wild boar bone tissues were sourced from PRJCA032946 [14].

### Quantitative real-time PCR and semi-quantitative PCR

PTVCs were lysed using TRNzol reagent (DP424, TIANGEN, Beijing, China) for total RNA extraction. To detect mRNA expression, 2 μg of total RNA from each sample was reverse-transcribed into cDNA using the FastKing RT Kit (with gDNase) (KR118, TIANGEN, Beijing, China) according to the manufacturer’s recommendations. Quantitative RT-PCR (qRT-PCR) was performed in 20 μL reactions containing 10 μL of 2 × Universal SYBR Green Fast qPCR Mix (RK21203, ABclonal, Wuhan, China), 8 μL RNase-free water, 0.5 μL each of forward and reverse primers (10 μmol/L), and 1 μL cDNA (~ 300 ng). The thermocycling conditions were: 95 °C for 15 min; 40 cycles of 94 °C for 20 s and 60 °C for 34 s. *HPRT1* was used as the internal reference. Primer sequences are shown in table S1. Semi-quantitative PCR used the same reaction mix and primer sequences but was run for 30 cycles. Products were separated on a 2% agarose gel and visualized using a gel imaging system.

### Immunohistochemistry of vertebral tissues

Immunohistochemistry (IHC) was performed using the standard protocol. Briefly, thoracic vertebra tissues of 3-day-old Yorkshire pigs, including intervertebral discs and the endplates of the upper and lower vertebrae, were collected. The surrounding muscle tissues were removed, and the tissues were rinsed with normal saline or PBS. The tissue samples were then placed in a fixative solution. Rabbit polyclonal primary antibody against RFLNA (Bioss, Beijing, China; 1:150 dilution). HRP-labeled goat anti-rabbit secondary antibody (Beyotime Biotechnology, Shanghai, China; 1:200 dilution) was then incubated at room temperature.

### Immunofluorescence of vertebral tissues

Immunofluorescence (IF) was carried out using the standard protocol. Briefly, tissue sections were treated with EDTA antigen retrieval buffer (pH 9.0), and HS antigen retrieval was performed by intermittent heating in a microwave oven. A spontaneous fluorescence quenching agent was dropped and incubated for 5 min. The primary anti-RFLNA (Bioss, Beijing, China) antibody solution was diluted at a ratio of 1:100 and incubated overnight at 4 °C. The secondary antibody solution was diluted at a ratio of 1:300 and incubated in the dark at room temperature for 1 h. DAPI staining solution was dropped and incubated in the dark for 10 min to label the cell nuclei. The tissues were fixed with an anti-fluorescence quenching mounting medium. The samples were observed and imaged using a fluorescence microscope.

### Plasmid construction

The coding sequence region sequence of porcine *RFLNA* was amplified from porcine total mRNA using the conventional PCR procedure. When designing the primers, enzyme recognition sequences were added to the upstream (Kpn Ⅰ) and downstream (Bgl Ⅱ) primers respectively. The upstream primer of *RFLNA* was F: 5′-GGGGTACCGCCACCATGGTGGGCCACCTGC-3′; the downstream primer was R: 5′-GAAGATCTTCAGAGCGCGGCAGGGCCCAGGAGG-3′. It was ligated into the pcDNA3.1-EGFP plasmid through a double digestion system to form the pcDNA3.1-*RFLNA*-EGFP gene expression plasmid. A small interfering RNA (siRNA) targeting *RFLNA* (sequence: 5′-CCUACAGCGAGACGAUCGUtt-3′) was synthesized by GenePharma Co., Ltd. (Shanghai, China).

### Cell transfection

The 2 mL of diluted PTVC cells (with a cell concentration of 5 × 10^5^ cells/mL) were seeded into the wells of a 12-well plate (2 mL per well) and cultured for 48 h. Subsequently, when the cell density reached 60%, Lipofectamine™ 3000 (Invitrogen, Carlsbad, CA, USA) was used to transfect 1 μg of plasmid or interference fragments (30 pmol) per well, and the transfection lasted for 48 h. The transfection process was carried out in RPMI-1640 medium containing 10% FBS.

### Cell scratch assay

On the back of the 6-well plate (Corning, NY, USA), 5 horizontal lines were drawn with a marker pen to divide each well into 6 parts. Cells were seeded into the 6-well plate. After the cells reached 100% confluence, a straight line was scratched vertically with a yellow pipette tip, applying the same force in each well. The floating cells were washed away with PBS, and the medium was replaced with serum-free medium. The migration of cells at the same position was photographed and recorded at 0, 6, 12, 24, and 48 h respectively.

### Cell adhesion

50 μL of diluted fibronectin (diluted to 10 μg/mL with serum-free medium) was added to each well of a 96-well plate (Corning, NY, USA), and it was air-dried overnight in a clean bench (*n* = 6). The coating solution was aspirated, and 200 μL of 1% BSA was added and the plate was incubated at 37 °C for 1 h. After the cells to be tested were treated, the cells were digested with 0.25% EDTA trypsin, and the concentration of the cell suspension was adjusted with serum-free medium. The cells were seeded into the 96-well plate at a density of 5 × 10^4^ cells per well with a volume of 100 μL per well. Meanwhile, blank control wells with only culture medium (without cells) were set. Five replicate wells were set for each group of cells, and the edge wells were filled with sterile PBS. The cells were placed in an incubator at 37 °C with 5% CO_2_ for 1 h. The cell culture plate was taken out, the medium was aspirated, and the wells were gently rinsed 3 times with PBS. 100 μL of fresh medium and 10 μL of CCK-8 solution (5 mg/mL) were added to each well, and the plate was incubated at 37 °C for 2 h. The absorbance of each well was measured at a wavelength of 490 nm using a microplate reader. The cell adhesion rate was calculated as follows: Cell adhesion rate = [(OD value of experimental group cells − OD value of blank)/(OD value of control group cells − OD value of blank)] × 100%.

### Transwell migration assay

Take PTVCs in good growth condition, digest and resuspend them. After mixing with 200 μL of serum-free medium, inoculate 4 × 10^4^ cells into the transwell chamber (Corning, NY, USA). Add 700 μL of medium containing 20% FBS to the lower culture chamber. Place it in the incubator and continue to culture for 24–48 h. Take out the culture plate, remove all the medium, wash it twice with PBS. Put the chamber into a new 24-well plate, add 600 μL of 4% paraformaldehyde (Solarbio, Beijing, China) to fix the cells for about 30 min, and then wash it 2–3 times with PBS. Stain with 1 × modified Giemsa staining solution (Beyotime Biotechnology, Shanghai, China) in the dark for 20 min, and wash it twice with PBS. Use a cotton swab to wipe off the cells that have not migrated in the chamber. Observe under a microscope and randomly select 3–5 fields of view for photographing.

### Statistical analysis

Statistical analysis was performed using GraphPad Prism 9.0. A nonpaired *t*-test was used to determine the significance between the two groups. Two-way analysis of variance (ANOVA) with Sidak's multiple comparisons test was used to determine the significance of differences between groups (^*^P < 0.05, significant; ^**^P < 0.01, highly significant; ns, no significant). Significance analysis of DEGs in RNA-seq was conducted using DESeq2 package in R. GraphPad Prism 9.0 was used for plotting the data, and all data were presented as mean ± SEM.

## Results

### HS inhibits the proliferation of PTVCs and the formation of the extracellular matrix

**Fig. 1:**
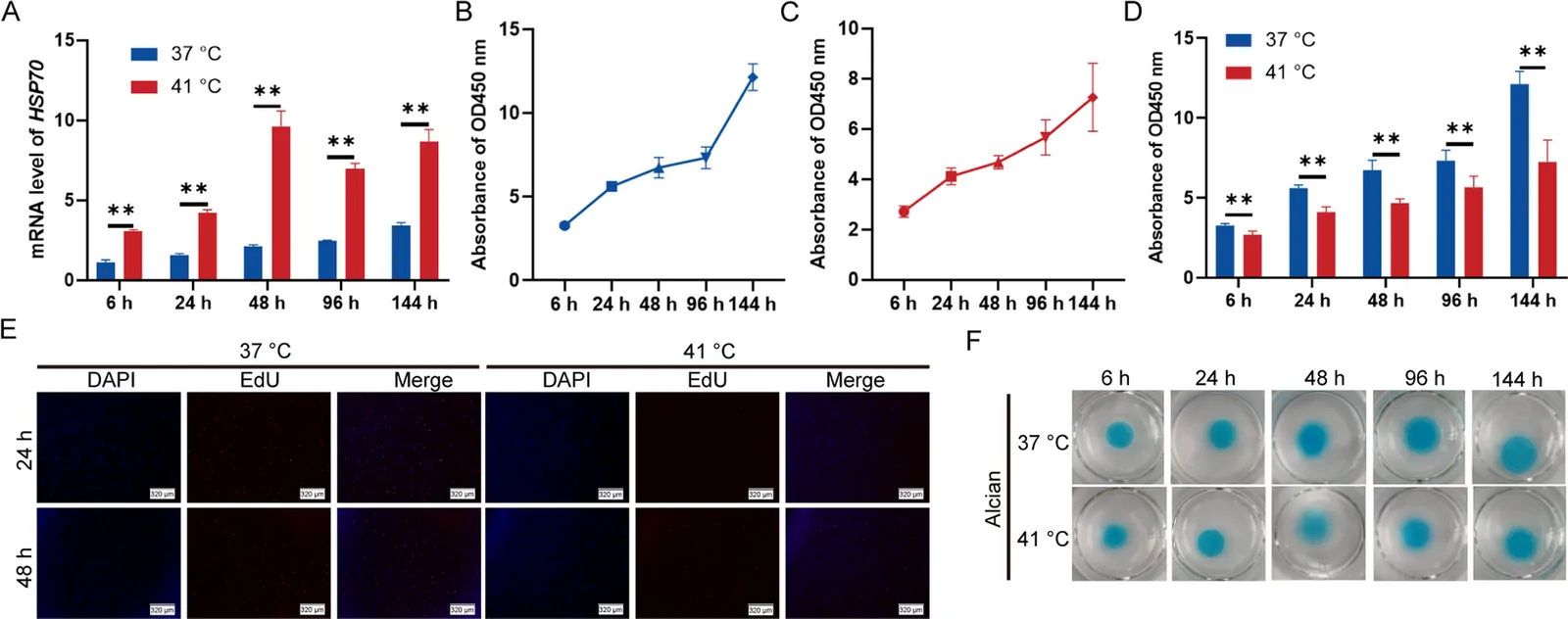
Effects of HS culture on PTVC proliferation and extracellular matrix formation. **A** The mRNA expression levels of the HS marker gene *HSP70* in PTVCs at different stages of differentiation induced by control-temperature (37 °C) and HS (41 °C). **B** Detection of CCK-8 cell proliferation of PTVCs at different stages of differentiation induced by 37 °C. **C** Detection of CCK-8 cell proliferation of PTVCs at different stages of differentiation induced by 41 °C. **D** The differences in CCK-8 cell proliferation of PTVCs at different stages of differentiation induced by 37 °C and 41 °C. **E** Verification of the proliferation of PTVCs at 24 h and 48 h of differentiation induced by 37 °C and 41 °C through EdU staining, with a scale of 320 μm. **F** Alcian blue staining of PTVCs at different stages of differentiation induced by 37 °C and 41 °C. Data are expressed as the mean ± SEM (CCK-8 *n* = 6; EdU *n* = 4). The experiment was repeated three independent times. ^**^P < 0.01

The HS response was robustly induced in PTVCs cultured at 41 °C, as evidenced by a significant (*P* < 0.01) upregulation of *HSP70* mRNA expression—a canonical HS marker—across all differentiation time points (6, 24, 48, 96, and 144 h) compared to the 37 °C control (Fig. 1A).

Under control-temperature, PTVC proliferation increased markedly over time, as confirmed by CCK-8 assays (Fig. 1B). Although cells continued to proliferate at 41 °C, the proliferation rate was markedly reduced (Fig. 1C). This suppression was evident at each stage of chondrogenic differentiation (*P* < 0.01, Fig. 1D). These findings were corroborated by EdU incorporation assays, which showed consistently inhibited proliferation under HS at 24 h and 48 h (Fig. 1E).

The impact of HS on extracellular matrix (ECM) synthesis was assessed via Alcian blue staining. At 37 °C, staining intensity deepened progressively with longer culture duration, accompanied by an increase in cell cluster size, indicating enhanced ECM accumulation (Fig. 1F). In contrast, under 41 °C conditions, Alcian blue staining was noticeably lighter and cell clusters were smaller, demonstrating suppression of ECM formation under HS (Fig. 1F).

### Analysis of the transcriptional profiles of PTVCs cultured at 37 °C for different durations

**Fig. 2:**
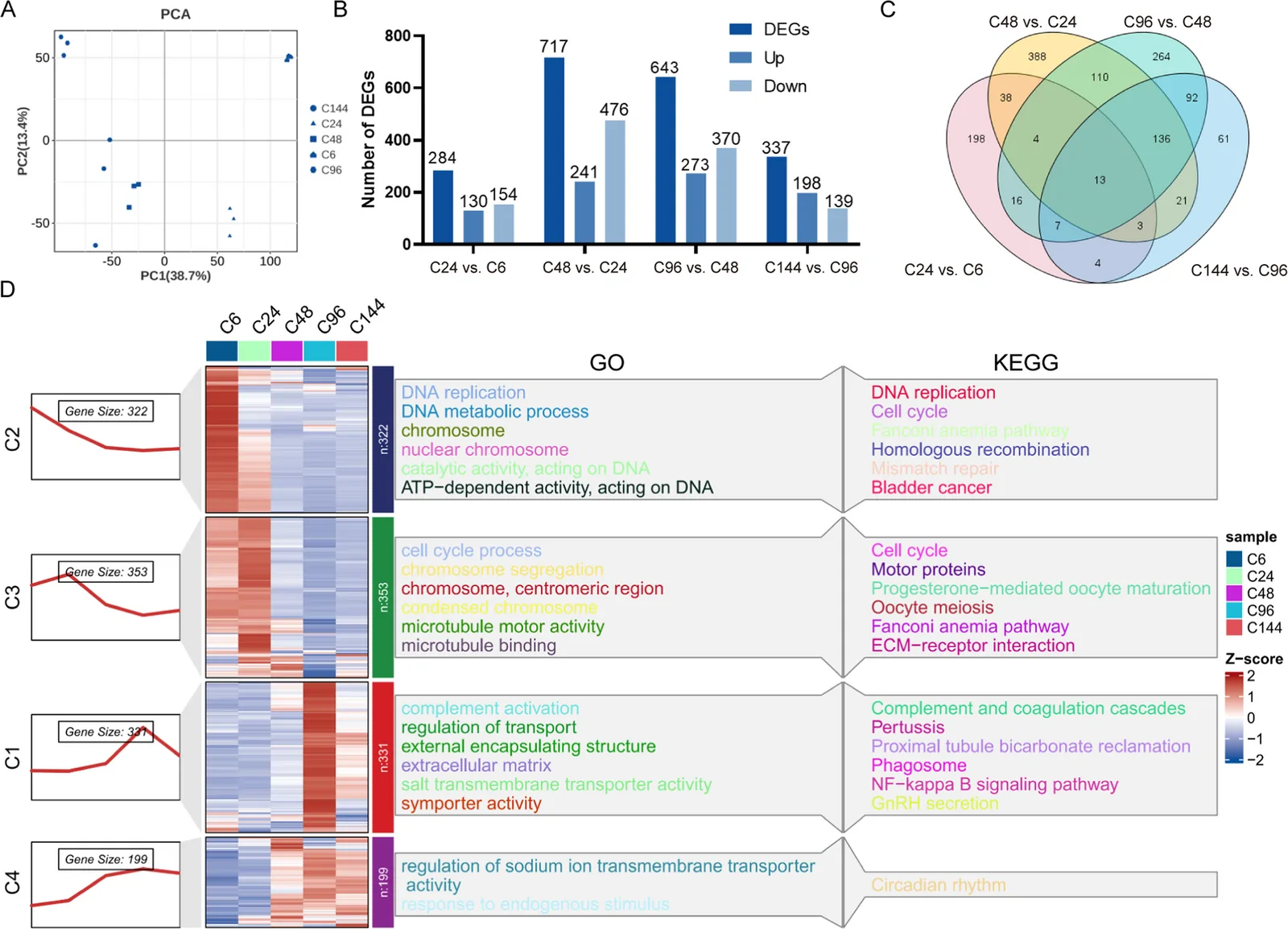
Identification, clustering, and functional enrichment analysis of DEGs at 37 °C for different culture durations. **A** PCA of samples in each group in transcriptomics. **B** The number of DEGs. **C** Venn diagram of DEGs. **D** Heatmap of the four clusters at five time points, and the top 5 significantly enriched GO pathways and KEGG pathways

Principal component analysis (PCA) of transcriptomes across five time points under 37 °C revealed distinct clustering, primarily along the first principal component (Fig. 2A). Comparison of adjacent differentiation stages (C24 vs. C6, C48 vs. C24, C96 vs. C48, and C144 vs. C96) identified 284, 717, 643, and 337 DEGs, respectively (Fig. 2B). DEG counts at 24 h, 48 h, and 96 h were significantly higher than other time points (Fig. 2B). Intersection analysis revealed 198, 388, 264, and 61 unique DEGs in these four comparison groups, respectively (Fig. 2C).

Functional enrichment analyses were performed for each group to characterize DEGs. In C24 vs. C6, GO analysis showed significant enrichment in biological processes (BP) such as the cellular response to fibroblast growth factor stimulation, cellular components (CC) associated with the extracellular region, and molecular functions (MF) including receptor ligand activity (Fig. S1A). KEGG analysis identified enrichment in the cytokine-cytokine receptor interaction pathway (Fig. S1B). For C48 vs. C24, GO analysis highlighted primary enrichment in BP related to the cell cycle, CC associated with chromosomes, and MF involving microtubule binding (Fig. S1C), with KEGG analysis confirming enrichment in cell cycle and motor protein signaling pathways (Fig. S1D). C96 vs. C48 DEGs were primarily enriched via GO in BP related to the cell cycle, CC associated with the extracellular region, and MF encompassing microtubule binding and cytoskeletal motor activity (Fig. S1E), with KEGG enrichment in cell cycle and motor protein pathways (Fig. S1F). In C144 vs. C96, GO analysis linked DEGs to cell cycle-related BP, condensed chromosome-associated CC, and microtubule/cytoskeletal motor activity-related MF (Fig. S1G), while KEGG analysis also indicated enrichment in cell cycle and motor protein pathways (Fig. S1H). Collectively, these results suggest DEGs mediate critical regulatory functions during PTVC differentiation, with pathways governing cell cycle and cytoskeletal dynamics being predominant.

Time-series cluster analysis categorized DEGs into four distinct expression profiles (Fig. 2D). Cluster 1 (337 DEGs) showed a predominant upward trend, peaking at 96 h, with GO enriched in complement activation, transport regulation, and KEGG enriched in complement-coagulation cascades, proximal tubule bicarbonate reabsorption. Cluster 2 (322 DEGs) exhibited a downward trend, GO enriched in DNA replication and metabolism, KEGG enriched in DNA replication/cell cycle pathways. Cluster 3 (353 DEGs) peaked at 24 h with an initial rise followed by decline, GO enriched in cell cycle regulation, chromosome segregation, and cytoskeletal development, and cell cycle, motor protein, and KEGG enriched in ECM-receptor interaction pathways. Cluster 4 (199 DEGs) displayed a progressively increasing pattern, GO enriched in sodium ion transport regulation, endogenous stimulus response, KEGG enriched in circadian rhythm signaling. These findings underscore the central role of the cell cycle and highlight the critical functional contribution of cytoskeletal proteins during PTVC differentiation.

### Analysis of the transcriptional profiles of PTVCs cultured at 41 °C for different durations

**Fig. 3:**
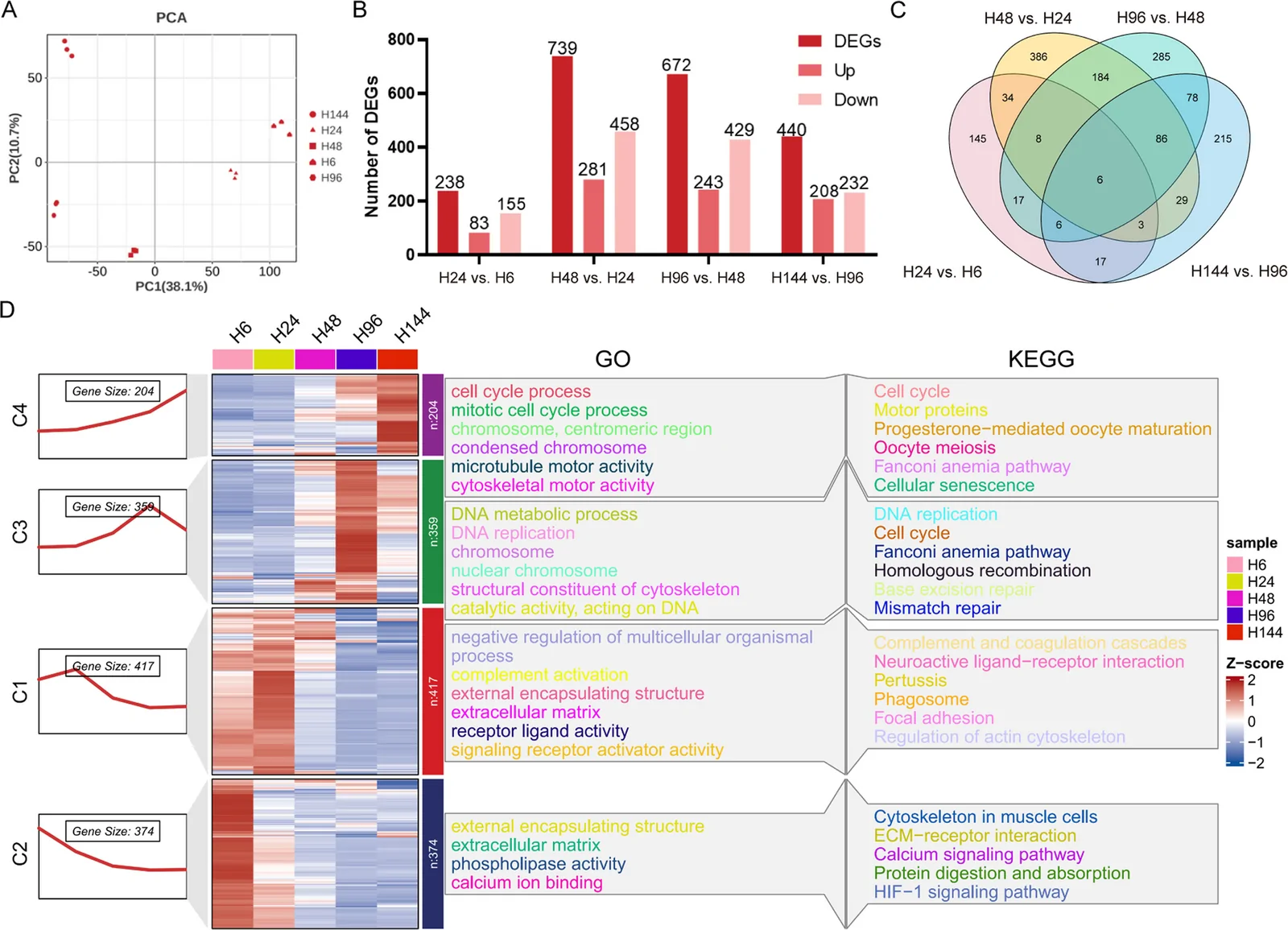
Identification, clustering, and functional enrichment analysis of DEGs at 41 °C for different culture durations. **A** PCA in transcriptomics. **B** The number of DEGs. **C** Venn diagram of DEGs. **D** Heatmap of the four clusters at five time points, and the top 5 significantly enriched GO pathways and KEGG pathways

PCA of the 41 °C HS groups also showed distinct clustering across time points (Fig. 3A). Analysis of adjacent stages (H24 vs. H6, H48 vs. H24, H96 vs. H48, H144 vs. H96) identified 238, 739, 672, and 440 DEGs, respectively (Fig. 3B). Consistent with control-temperature group, DEGs counts at 24 h, 48 h, and 96 h were significantly higher than other time points (Fig. 3B). Intersection analysis revealed 145, 386, 285, and 215 unique DEGs in these groups (Fig. 3C).

For H24 vs. H6, DEGs were enriched in BP related to morphogenesis during developmental growth, CC associated with the extracellular region, and MF including signal receptor binding (Fig. S2A). KEGG analysis identified enrichment in the MAPK signaling and cytokine-cytokine receptor interaction pathways (Fig. S2B). For H48 vs. H24, GO analysis showed primary enrichment in BP related to the cell cycle, CC associated with chromosomes, and MF involving tubulin binding and cytoskeletal motor activity (Fig. S2C). KEGG analysis confirmed enrichment in cell cycle and motor protein signaling pathways (Fig. S2D). In H96 vs. H48, GO analysis indicated DEGs were enriched in BP including the cell cycle, CC associated with the extracellular region, and MF encompassing signaling receptor binding, microtubule binding, and cytoskeletal motor activity (Fig. S2E). KEGG analysis revealed similar enrichment in cell cycle and motor protein pathways (Fig. S2F). For H144 vs. H96, GO analysis linked DEGs to systemic processes BP, the extracellular region CC, and signal receptor binding MF (Fig. S2G), while KEGG analysis highlighted enrichment in the calcium signaling pathway, muscle cytoskeleton, cell cycle, and motor protein pathways (Fig. S2H).

Time-series clustering under HS also yielded four expression profiles (Fig. 3D). Cluster 1 (417 DEGs) exhibited an initial increase followed by a continuous decline, peaking at 24 h; GO analysis showed enrichment in negative regulation of multicellular processes, complement activation, and external coated structures, while KEGG analysis identified the complement-coagulation cascade, neuroactive ligand-receptor interaction, and regulation of actin cytoskeleton pathways. Cluster 2 (374 DEGs) displayed a gradual downward trend, GO analysis showed enrichment in external encapsulating structures, extracellular matrix, and phospholipase activity, with KEGG pathways including muscle cytoskeleton-ECM interaction, calcium signaling, and HIF-1 signaling. Cluster 3 (359 DEGs) peaked at 96 h with an initial rise followed by decline, GO enrichment in DNA metabolism, replication, and cytoskeletal structural components, KEGG pathways including DNA replication/cell cycle pathways. Cluster 4 (204 DEGs) showed a predominant upward trend, peaking at 144 h, with GO enrichment in cell cycle regulation, mitotic processes, and cytoskeletal motor activity, and KEGG enrichment in cell cycle and motor protein pathways. This analysis confirms that differentiation under HS is likewise governed by pathways associated with the cell cycle and cytoskeletal remodeling.

### Joint analysis of transcriptome data of PTVCs cultured at 37 °C and 41 °C for different durations

**Fig. 4:**
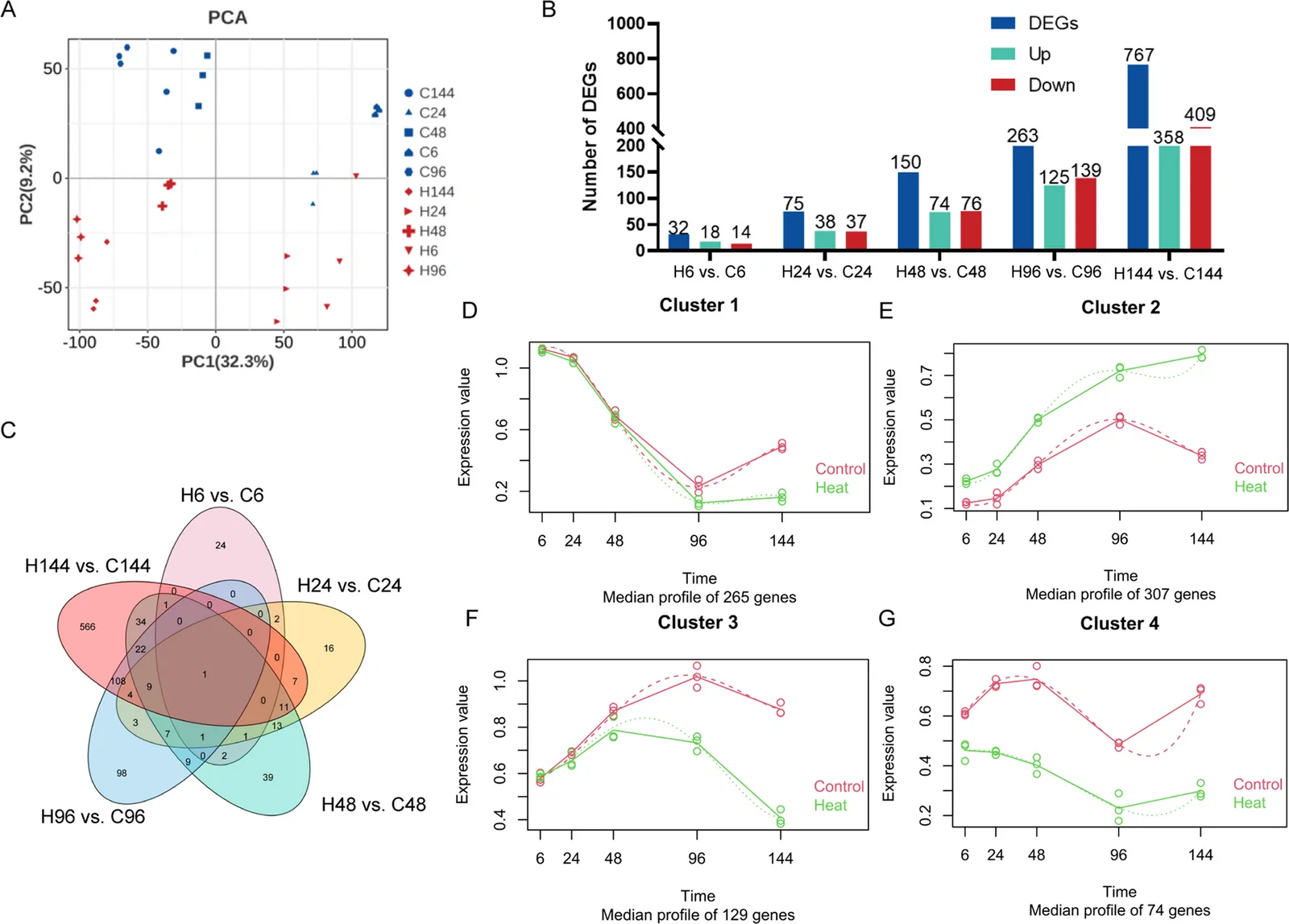
Identification, clustering, and functional analysis of DEGs between 41 °C and 37 °C cultures at different time points. **A** PCA in transcriptomics. **B** The number of DEGs. **C** Venn diagram of DEGs. **D** Cluster 1 in the time-series clustering. **E** Cluster 2 in the time-series clustering. **F** Cluster 3 in the time-series clustering. **G** Cluster 4 in the time-series clustering

Combined analysis of matched time points (H6 vs. C6, H24 vs. C24, H48 vs. C48, H96 vs. C96, H144 vs. C144) revealed clear separation between control and HS groups (Fig. 4A). Using 24 h and 48 h as boundaries, the five data pairs partitioned into two clusters, consistent with prior analyses, indicating that 24–48 h marks a critical transition for cartilage proliferation and differentiation (Fig. 4A). We identified 32, 75, 150, 263, and 767 DEGs in the five comparison groups, respectively (Fig. 4B). The number of DEGs increased with longer HS exposure, indicating a cumulative cellular impact. Intersection analysis revealed 24, 16, 39, 98, and 566 unique DEGs in each respective comparison (Fig. 4C).

GO analysis of H6 vs. C6 DEGs revealed significant enrichment in BP such as negative regulation of signal transduction, CC associated with the mitochondrial proton-transporting ATP synthase complex, and MF including zinc/transition metal ion binding (Fig. S3A). KEGG enrichment in the RIG-like receptor signaling pathway, IL-17 signaling pathway, and NF-kappa B signaling pathway (Fig. S3B). For H24 vs. C24, GO analysis highlighted BP related to viral/symbiont defense responses, CC encompassing the extracellular region and actin cytoskeleton, and MF including receptor-ligand activity (Fig. S3C), with KEGG analysis showing enrichment in the MAPK signaling pathway, hepatitis C, and influenza A (Fig. S3D). H48 vs. C48 DEGs were enriched in BP involving biotic stimulus/symbiont defense responses, CC associated with the extracellular region, and MF such as transmembrane signal receptor activity (Fig. S3E). KEGG analysis identified enrichment in neuroactive ligand-receptor interaction, ECM-receptor interaction, and ABC transporter pathways (Fig. S3F). H96 vs. C96 DEGs showed BP enrichment in cell population proliferation, CC related to the extracellular region, and MF including signal receptor/receptor-ligand activity (Fig. S3G), with KEGG enrichment in muscle cytoskeleton and chemical carcinogenesis-receptor activation pathways (Fig. S3H). H144 vs. C144 DEGs were enriched in BP such as cell population proliferation, CC associated with the extracellular region, and MF including signal receptor binding/regulatory activity (Fig. S3I), while KEGG analysis highlighted muscle cytoskeleton, PI3K-AKT, cell cycle, motor protein, and ECM-receptor interaction pathways (Fig. S3J). This pattern indicates HS affects chondrocyte development through pathways involving extracellular components, the cell cycle, and cytoskeletal proteins.

Integrated time-series analysis identified 775 co-expressed DEGs across temperatures, clustered into four profiles (Fig. 4D–G). Cluster 1 (265 DEGs) exhibited a downward expression trend, with higher control-temperature expression, enriched in BP such as cell cycle/mitotic processes, CC including microtubule cytoskeleton/chromosomal centromeric region, and MF such as tubulin/motor activity (Fig. S4A and B). Cluster 2 (307 DEGs) showed an upward trend with higher HS expression, enriched in BP like cell proliferation/lipid transport, CC such as external coated structures/ECM, and MF including signal receptor regulatory activity (Fig. S4C and D). Cluster 3 (129 DEGs) displayed an initial increase followed by decrease, with higher control-temperature expression, enriched in BP involving MAPK activity regulation, CC related to external coated structures/ECM, and MF such as calcium ion binding (Fig. S4E and F). Cluster 4 (74 DEGs) showed a V-shaped trend, enriched in BP like defense responses to viral, CC including neuron projection/presynaptic membrane, and MF such as cytoskeletal protein binding, with KEGG enrichment in hepatitis C, infuenza A, RIG-I-like receptor signaling pathways (Fig. S4G and H).

### Joint analysis of transcriptome data at cellular and tissue levels identified *RFLNA* as a key candidate gene involved in bone development

**Fig. 5:**
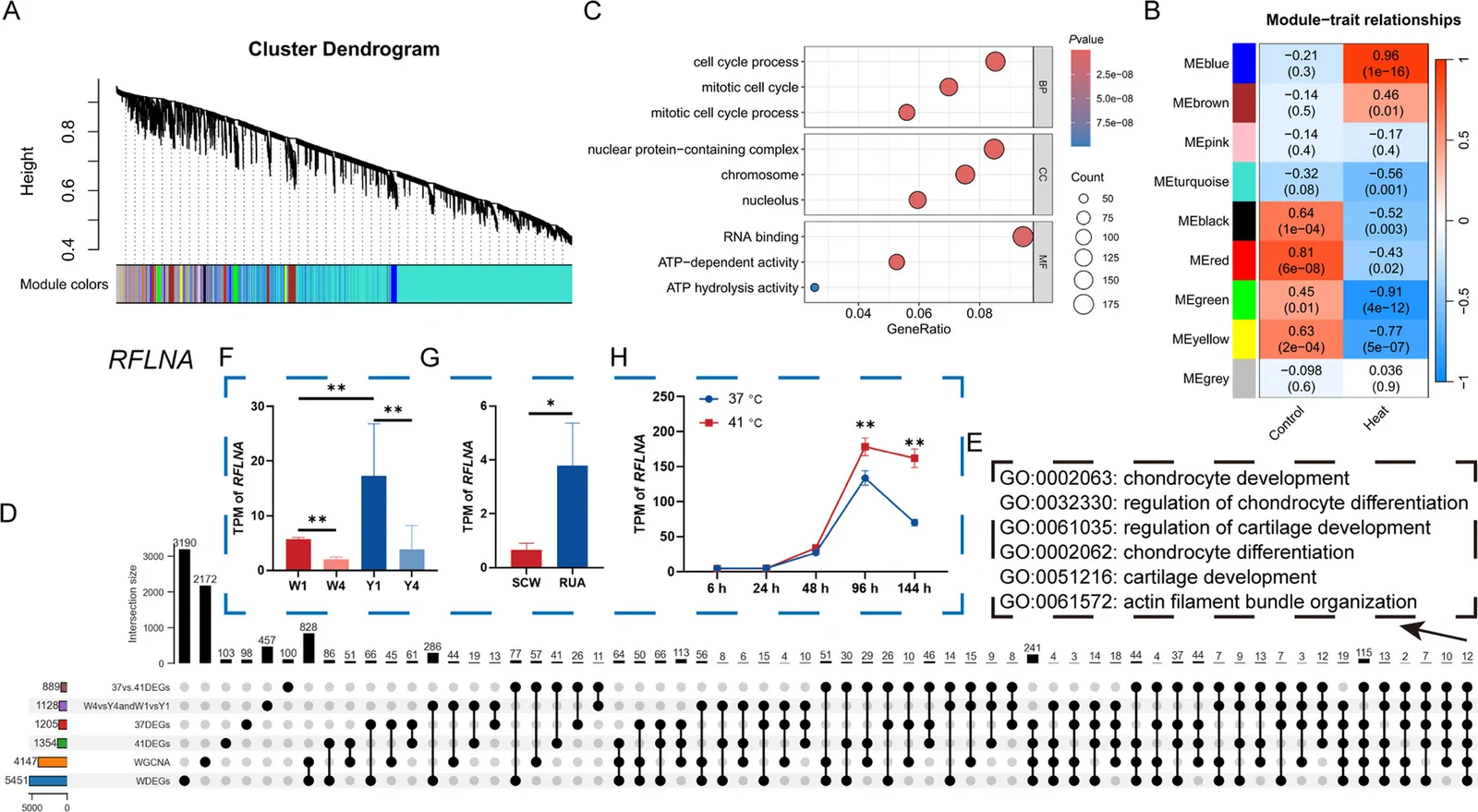
Integrated analysis of DEGs across temperature groups using WGCNA and multi-level transcriptome data. **A** Hierarchical clustering dendrogram from WGCNA identifying nine co-expression modules, each represented by a distinct color. **B** Module-trait relationship heatmap illustrating associations between module eigengenes (rows) and treatment groups (columns). **C** GO enrichment analysis of genes in modules significantly positively correlated with HS treatment. **D** Upset plot depicting the intersection of 6 datasets: DEGs from control vs. HS time-course comparisons, stage-specific DEGs under control/HS, WGCNA heat-associated hub genes, thoracic vertebra chondrocyte DEGs in 1-/4-month-old Wuzhishan/Yorkshire pigs, and bone tissue DEGs between low-temperature (RUA) and high-temperature (SCW) wild boars. **E** GO terms related to bone development enriched among the 12 genes common to all six datasets. **F** Transcriptome TPM expression of *RFLNA* in thoracic vertebra growth plate chondrocytes of 1- and 4-month-old Wuzhishan and Yorkshire pigs. **G** TPM expression of *RFLNA* in bone tissues of RUA and high-temperatureSCW wild boars. **H** TPM expression of *RFLNA* in PTVCs cultured for 6, 24, 48, 96, and 144 h under 41 °C and 37 °C. Data are expressed as the mean ± SEM (*n* = 3). *P < 0.05, **P < 0.01

WGCNA of gene expression across all samples identified nine modules (Fig. 5A). Module-trait correlation analysis revealed seven modules significantly associated (*P* < 0.05) with the 41 °C treatment, including two positive (blue, brown) and five negative (yellow, green, red, black, turquoise) correlations (Fig. 5B). Genes with strong module membership (|MM| > 0.8) and trait significance (|GS| > 0.2) were selected as core module genes, yielding 4,147 candidates. GO analysis revealed these genes were enriched in BP such as cell cycle regulation and mitotic progression, cellular components including nucleoprotein complexes and chromosomes, and molecular functions such as RNA binding (Fig. 5C). These modules reveal that HS mainly disrupts the normal process of cell development.

An integrated analysis was conducted across multiple datasets: DEGs from control vs. HS cultures, WGCNA core genes, maSigPro results, thoracic vertebra chondrocyte DEGs in 1- and 4-month-old Wuzhishan/Yorkshire pigs, and bone tissues of wild boars from low-temperature regions in Asia (RUA) and wild boars from high-temperature regions in southern China (SCW). Twelve genes were identified as common across all datasets (Fig. 5D). Among these, *RFLNA* was significantly enriched in skeletal development GO terms (Fig. 5E).

Transcriptome analysis of pig breeds showed higher *RFLNA* expression in 1-month-old Yorkshire pigs compared to Wuzhishan pigs, with both breeds exhibiting decreased expression at 4 months (*P* < 0.01, Fig. 5F). Its placement in a temperature-negatively correlated WGCNA module suggested a potential role in countering HS effects. Wild boars from low-temperature regions also displayed significantly higher *RFLNA* expression in bone tissues than high-temperature counterparts (*P* < 0.05, Fig. 5G). In vitro, *RFLNA* expression increased with chondrocyte development in both temperature groups, with significantly higher levels in HS cultures after 96 h (*P* < 0.01, Fig. 5H). These findings indicate *RFLNA* influences chondrocyte development, responds to HS, and shows differential expression correlated with body size and temperature, marking it as a key candidate for regulating vertebral development and heat adaptation.

### The expression pattern of RFLNA

**Fig. 6:**
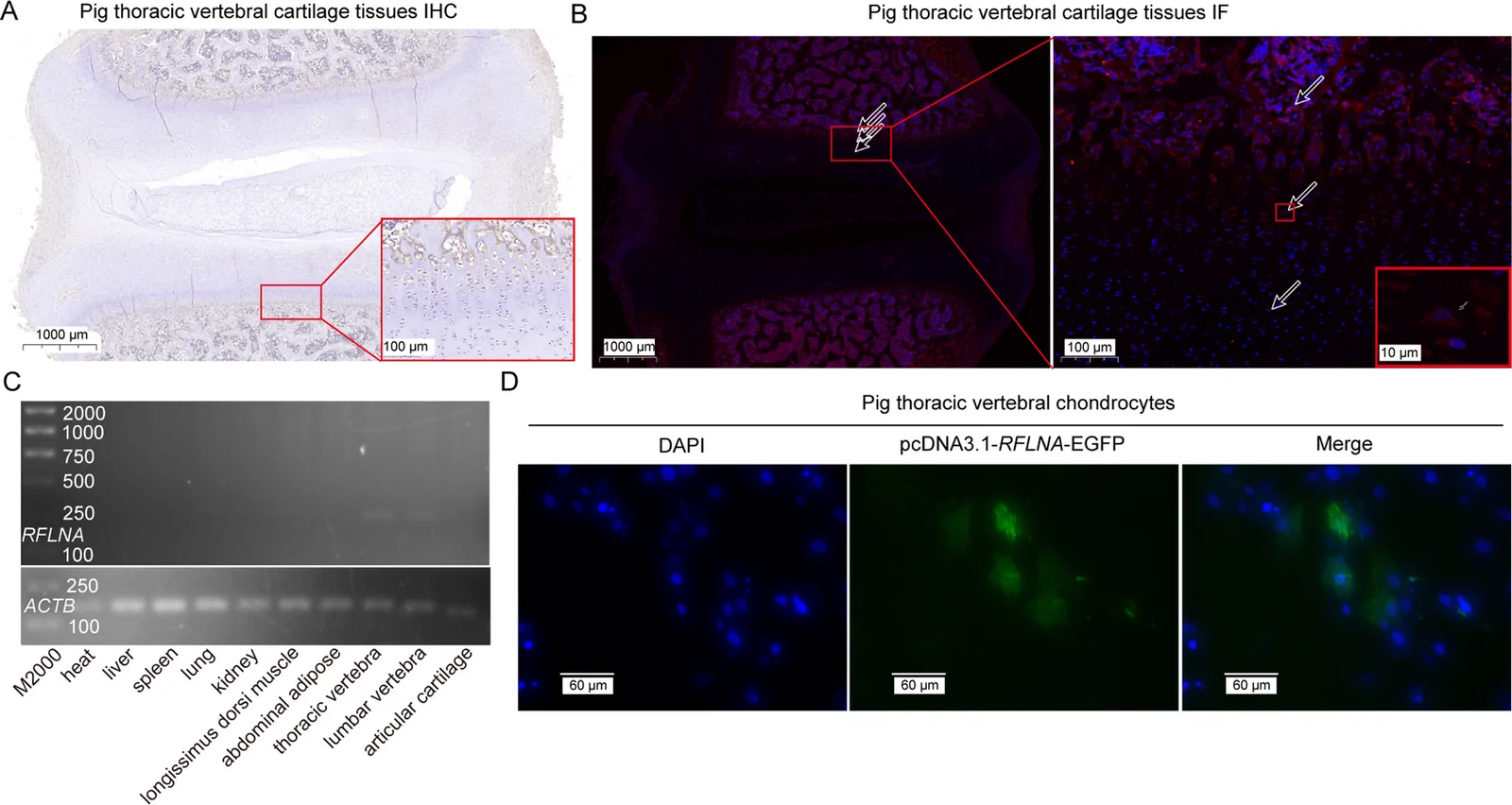
Expression pattern analysis of the *RFLNA* gene. **A** IHC of RFLNA in paraffin-embedded porcine vertebra sections, demonstrating protein localization in chondrocytes and osteocytes. **B** IF of RFLNA. **C** Semi-quantitative RT-PCR analysis of *RFLNA* expression in multiple tissues of 1-month-old Yorkshire pigs, including heart, liver, spleen, lung, kidney, longissimus dorsi muscle, abdominal adipose, thoracic vertebra, lumbar vertebra, and articular cartilage. **D** Subcellular localization of the pcDNA3.1-*RFLNA*-EGFP fusion protein in primary porcine thoracic vertebra chondrocytes, visualized by confocal microscopy

Serial sections of porcine vertebra tissues were subjected to RFLNA immunohistochemical staining (Fig. 6A). Concurrent immunofluorescence staining revealed subcellular localization of RFLNA protein within chondrocytes and osteocytes of porcine vertebrae (Fig. 6B), confirming its presence in both cartilage and bone cell lineages. Semi-quantitative RT-PCR across multiple tissues revealed highest *RFLNA* mRNA levels in thoracic and lumbar vertebrae, significantly exceeding levels in heart, liver, spleen, lung, kidney, muscle, fat, or articular cartilage (Fig. 6C). Transient expression of a pcDNA3.1-*RFLNA*-EGFP fusion construct in PTVCs demonstrated that RFLNA protein primarily localizes to the cytoplasm, displaying a fibrous pattern consistent with cytoskeletal association (Fig. 6D). These findings suggest that *RFLNA*, as a cytoskeletal protein-binding factor, exerts critical functions in the cytoplasm of growth plate chondrocytes, potentially mediating cytoskeletal dynamics during vertebral development or stress responses.

### *RFLNA* overexpression enhances proliferation and adhesion while attenuating migration of PTVCs

**Fig. 7:**
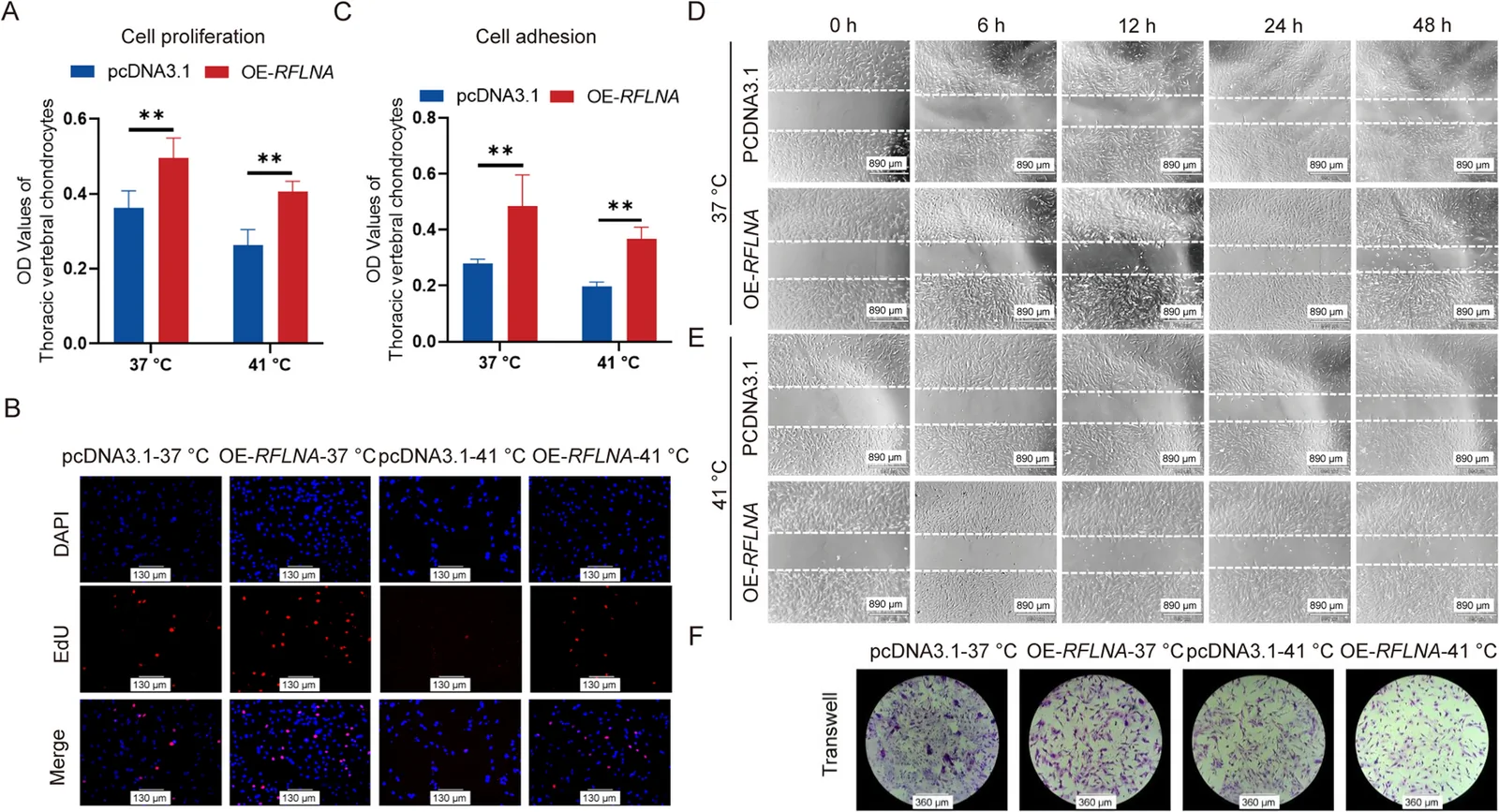
The effects of *RFLNA* overexpression on PTVC proliferation, adhesion and migration. **A** CCK-8 assay for cell proliferation after 48 h of culture under 37 °C and 41 °C temperatures. **B** EdU incorporation assay corroborating proliferation changes in PTVCs under both temperature conditions for 48 h. **C** Cell adhesion assay demonstrating enhanced adhesion capacity following *RFLNA* overexpression after 48 h of culture under 37 °C and 41 °C. **D** and **E** Cell scratch wound healing assays assessing migration dynamics at 0, 6, 12, 24, and 48 h under 37 °C and 41 °C conditions. **F** Transwell migration assay verifying reduced migratory potential in *RFLNA*-overexpressing PTVCs after 48 h of culture in both temperature environments. Data are expressed as the mean ± SEM (CCK-8 *n* = 6; EdU *n* = 4). The experiment was repeated three independent times. ^**^P < 0.01

Transfection efficiency for the *RFLNA* overexpression construct was confirmed by qPCR (Fig. S5A). Given the link between cytoskeletal remodeling and cell dynamics, we investigated *RFLNA*'s functional role. CCK-8 assays showed that *RFLNA* overexpression significantly enhanced PTVC proliferation under both 37 °C and 41 °C conditions (*P* < 0.01, Fig. 7A). This pro-proliferative effect was corroborated by EdU assays (Fig. 7B). Furthermore, *RFLNA* overexpression significantly increased cell adhesion at both temperatures (*P* < 0.01, Fig. 7C). Conversely, scratch wound assays revealed that HS attenuated cell migration compared to control conditions (Fig. 7D and E). Under 37 °C, *RFLNA* overexpression inhibited cell migration at 48 h (Fig. 7D). This anti-migratory effect was more pronounced under HS, where overexpression significantly reduced migratory capacity (Fig. 7E). Transwell migration assays validated these results, showing that 48 h of heat treatment decreased PTVCs migratory potential, and *RFLNA* overexpression further attenuated migration in both temperature groups (Fig. 7F).

### *RFLNA* knockdown impairs proliferation and adhesion while enhancing migration of PTVCs

**Fig. 8:**
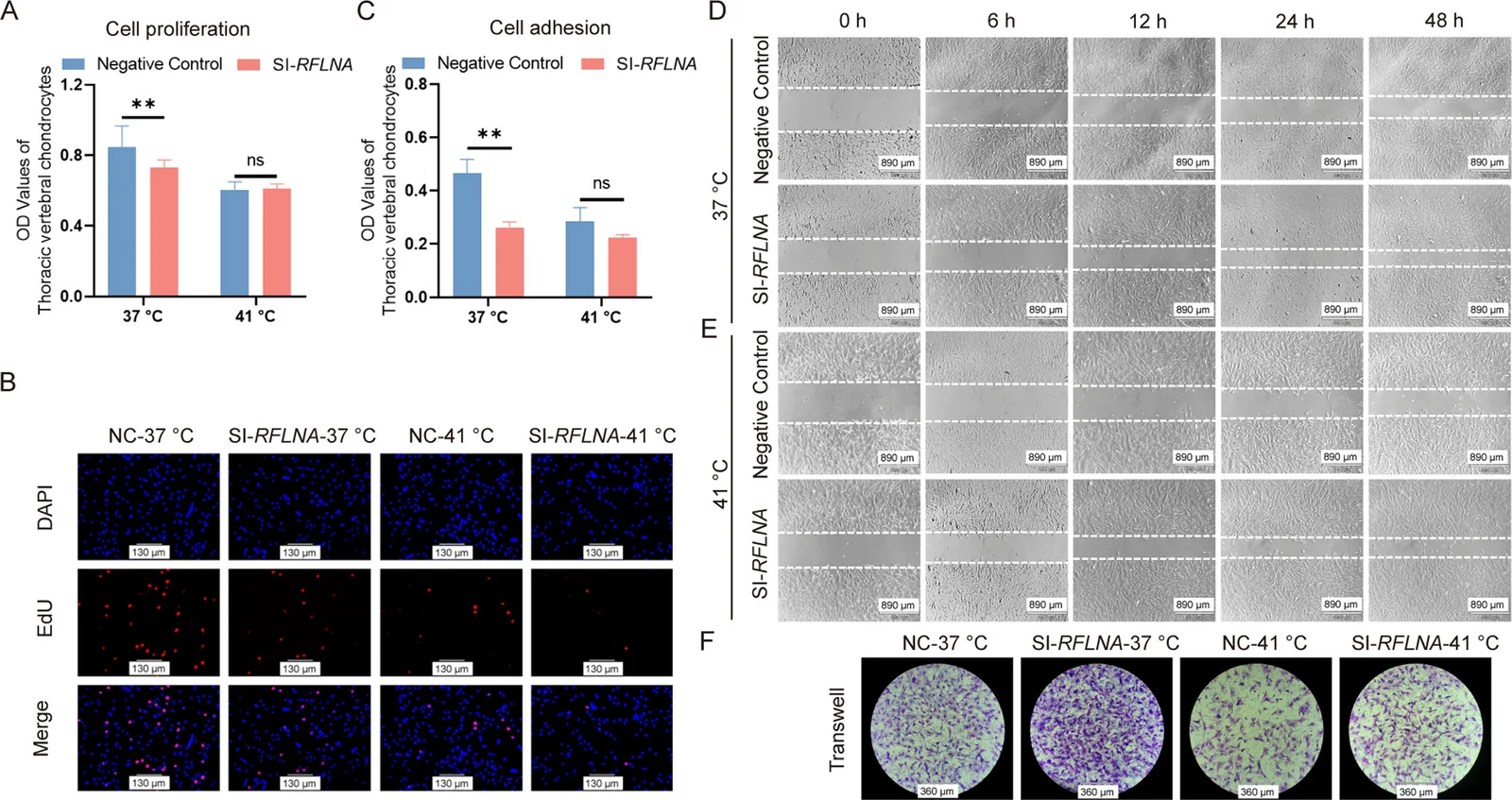
The effects of *RFLNA* knockdown on PTVC proliferation, adhesion and migration. **A** CCK-8 assay for cell proliferation after 48 h of culture 37 °C and 41 °C, showing impaired proliferation following *RFLNA*-knockdown (SI-*RFLNA*) in control conditions. **B** EdU incorporation confirmed a decrease in proliferation in *RFLNA*-knockdown PTVCs relative to the negative control (NC) after 48 h culture. **C** Cell adhesion assay in *RFLNA*-knockdown cells after 48 h under 37 °C and 41 °C culture. Cell scratch wound healing assays assessing migration dynamics at 0, 6, 12, 24, and 48 h in **D** 37 °C and **E** 41 °C. **F** Transwell migration assay verifying increased migratory capacity in *RFLNA*-knockdown PTVCs after 48 h of culture in both temperature environments. Data are expressed as the mean ± SEM (CCK-8 *n* = 6; EdU *n* = 4). The experiment was repeated three independent times. ^**^P < 0.01; ns, no significant

Knockdown efficiency was verified by qPCR, with fragment *RFLNA*-Sus-549 selected for subsequent experiments (Fig. S5B). CCK-8 assays showed that *RFLNA* knockdown significantly impaired PTVC proliferation under 37 °C after 48 h (*P* < 0.01), with no significant difference observed in proliferative capacity under 41 °C (Fig. 8A). EdU incorporation assays corroborated these results, showing reduced proliferation in knockdown cells under control-temperature, but not under HS (Fig. 8B). Similarly, knockdown significantly reduced cell adhesion at 37 °C (*P* < 0.01), but not under HS (Fig. 8C). In contrast, scratch assays demonstrated that *RFLNA* knockdown enhanced cell migration at all time points under 37 °C (Fig. 8D), an effect that was amplified under 41 °C (Fig. 8E). Transwell assays confirmed that knockdown increased PTVC migration in both conditions, with a more potent effect under HS (Fig. 8F).

## Discussion

HS disrupts normal cellular physiology and impairs proliferative capacity. For example, 41 °C culture significantly inhibits the proliferation of porcine skeletal muscle satellite cells [15], delays chondrocyte differentiation in the chicken tibia growth plate [16], and reduces the size of in vitro-cultured porcine articular chondrocyte pellets compared to 37 °C [17]. Consistent with these reports, our in vitro study elucidates that 41 °C profoundly suppresses the proliferation of PTVCs. However, it should be noted that our model primarily reflects direct thermal effects on chondrocytes in a controlled setting, and does not capture the potential modulation by systemic factors (e.g., hormones, metabolism) present in the whole animal under HS. Environmental temperature fluctuations in production settings directly impact swine growth [18]. Chinese indigenous pigs exhibit an inverse correlation between body length and latitude, with shorter body lengths observed at lower latitudes, which may be associated with higher annual average temperatures in these regions [19]. These results highlight that environmental temperature fluctuations in farming settings directly impact porcine growth, potentially influencing chondrocyte proliferation/differentiation and ultimately determining pig body length traits.

The fourth day represents a critical time point for porcine chondrocyte differentiation [20]. Consistent with this study, transcriptome sequencing revealed a significant increase in DEGs in the period from 24 to 96 h, identifying this as a pivotal window for cartilage development. These DEGs were predominantly enriched in functions related to cell cycle regulation, extracellular matrix organization, and microtubule binding—processes essential for maintaining chondrocyte proliferation, differentiation, and morphological integrity [21–23]. Notably, the cell cycle pathway ensures orderly cell division, while extracellular region-related functions and receptor-ligand interactions are pivotal for tissue patterning during differentiation.

Functional annotation of HS DEGs revealed enrichment not only in cell cycle and cytoskeletal pathways but also in stress-responsive processes, such as developmental morphogenesis and viral defense responses. KEGG analysis highlighted activation of the MAPK signaling and cytokine-cytokine receptor interaction pathways, consistent with HS-mediated activation of stress-response cascades (e.g., MAPK pathway-mediated attenuation of heterochromatin epigenetic modification in fission yeast [24]). These data indicate that while cells allocate resources to mitigate HS, they simultaneously endeavor to sustain core developmental programs. However, this balancing act involves altered regulatory mechanisms that may divert energy from normal proliferation and differentiation trajectories.

Integrated analysis of transcriptomes across temperature groups showed clear separation between control and HS conditions, with 24–48 h demarcating a critical transition point. The increasing number of DEGs with prolonged heat exposure underscores the cumulative cellular impact of chronic HS. Cross-population analyses that integrated data from different pig breeds and wild boar populations across distinct thermal environments consistently identified *RFLNA* as a key candidate gene significantly enriched in bone development-related GO terms. This aligns with equine studies linking *RFLNA* to withers height via selection signature analysis [25], thereby supporting its evolutionarily conserved role in regulating skeletal growth. It should be noted that the transcriptomic data in this study were obtained from three biological samples, which meets the minimum requirements for sequencing but limits the capture of broader genetic variability. Future studies should expand the sample size to improve the generalizability of the identified transcriptional signatures.

The *RFLNA*-encoded protein localizes to the cytoplasm, consistent with its proposed interaction with cytoskeletal components such as filamin A (*FLNA*) and filamin B (*FLNB*) to maintain cytoskeletal architecture [26]. In epiphyseal growth plate chondrocytes, the expression of mutant *FLNB* leads to chondrodysplasia [27]. As a scaffolding protein, *RFLNA* interacts with proteins such as *FLNB* and plays an important role in the assembly and functional maintenance of the cytoskeleton [9]. During growth plate ontogeny, chondrocytes transition from a random transverse organization in the resting and early proliferation zones—characterized by round/oval morphology and loose distribution—to an ordered longitudinal columnar arrangement in the late proliferation and hypertrophy zones, forming a distinctive stacked pattern along the long bone axis [28, 29]. This morphological remodeling is tightly coupled with cell proliferation, matrix secretion, and mechanical cues, with cytoskeletal proteins serving as key regulators [30–33]. For instance, Rho GTPases mediate actin reorganization to direct cellular elongation [34]. As a cytoskeletal scaffolding protein, *RFLNA* interacts with partners such as *FLNB*, playing a significant role in cytoskeletal construction and functional maintenance [9]. In this study, functional assays revealed novel roles for *RFLNA* in chondrocyte behavior: overexpression promoted proliferation and adhesion while inhibiting migration in both 37 °C and 41 °C environments, whereas knockdown had opposing effects. Notably, prolonged 41 °C culture upregulated *RFLNA* expression, and GO enrichment linked it to regulation of chondrocyte differentiation. WGCNA further demonstrated its membership in a module negatively correlated with temperature, suggesting a protective function against heat-induced proliferative inhibition. These compelling in vitro data establish *RFLNA* as a key mediator; however, the proposed protective function requires validation in more complex, physiologically relevant models. Our monolayer culture system is important for dissecting cell-autonomous mechanisms, yet it cannot fully recapitulate the intricate three-dimensional architecture, mechanical loading, and systemic signaling of the intact growth plate in vivo—a challenge underscored by the fact that no functional growth plate analogue has yet been engineered [35].

Collectively, our findings establish *RFLNA* as a pivotal mediator of the chondrocyte response to HS. Its upregulation appears to be a compensatory mechanism through which cells counteract heat-induced proliferation inhibition. This represents a newly identified target for potentially mitigating HS-impaired cartilage development. While this discovery advances our understanding of temperature-dependent skeletal growth regulation and provides a theoretical foundation for breeding heat-resilient swine, translating this potential into application necessitates future work that addresses the current limitations: elucidating the intracellular signaling mechanisms of *RFLNA* and, crucially, validating its in vivo relevance and regulatory networks to directly inform precision breeding strategies for climate-resilient swine production.

## Conclusions

In conclusion, HS triggers an acute cellular stress response and subsequently inhibits chondrocyte proliferation. Our study demonstrates that chondrocytes may resist HS-induced growth inhibition by upregulating *RFLNA*, which could serve as a potential target for alleviating HS-impaired cartilage development. These findings reveal that *RFLNA* links thermal stress responses to cytoskeletal regulation in chondrocytes, providing new molecular insights into how environmental temperature interacts with genetic factors to shape porcine body conformation traits.

## Supplementary Information

Additional file 1: Table S1. Primer sequences of genes selected for qRT-PCR.

Additional file 2: Fig. S1. Functional enrichment analyses of DEGs at distinct stages under 37 °C. Fig. S2. Functional enrichment analyses of DEGs at distinct stages under 41 °C culture. Fig. S3. Functional enrichment analyses of DEGs from 41 °C vs. 37 °C comparisons at corresponding time points. Fig. S4. Functional enrichment analyses of DEGs from 41 °C vs. 37 °C comparisons in the time-series clustering. Fig. S5. Detection of *RFLNA* overexpression and interference efficiency and expression of related marker genes.

Additional file 3. Semi-quantitative RT-PCR analysis of RFLNA expression in multiple tissues of 1-month-old Large White pigs, including heart, liver, spleen, lung, kidney, longissimus dorsi muscle, abdominal adipose, thoracic vertebra, lumbar vertebra, and articular cartilage.

## Data Availability

The data for the current study are available from the corresponding author upon reasonable request.

## Abbreviations

CCK8: Cell Counting Kit-8
cDNA: Complementary DNA
DEGs: Differentially expressed genes
DMEM-F12: Dulbecco’s modified Eagle’s medium F12
EdU: 5-Ethynyl-2-deoxyuridine
FBS: Fetal bovine serum
GO: Gene Ontology
HS: Heat stress
KEGG: Kyoto Encyclopedia of Genes and Genomes
PTVCs: Porcine thoracic vertebral growth plate chondrocytes
qRT-PCR: Quantitative real-time PCR
RNA-seq: High-throughput sequencing of RNA
TPM: Transcripts per million
WGCNA: Weighted Gene Co-expression Network Analysis
