# Correction: RFLNA mitigates heat stress-impaired chondrocyte proliferation and vertebral development through cytoskeletal regulation in pigs

**DOI:** 10.1186/s40104-026-01518-4

**Published:** 2026-10-14

**Authors:** Xiaoyang Yang, Yuxuan Xie, Yabiao Luo, Yubei Wang, Lixian Yang, Longmiao Zhang, Pengxiang Xue, Chengwan Zha, Meiying Fang

**Affiliations:** 1 State Key Laboratory of Animal Biotech Breeding, National Engineering Laboratory for Animal Breeding, MOA Key Laboratory of Animal Genetics and Breeding, College of Animal Science and Technology, Frontiers Science Center for Molecular Design Breeding, Beijing Key Laboratory for Animal Genetic Improvement, China Agricultural University, Beijing, 100193 China; 2 Sanya Institute of China Agricultural University, Sanya, 572000 China

**Correction: J Animal Sci Biotechnol 17, 68 (2026)**

**https://doi.org/10.1186/s40104-026-01387-x**

Following the publication of original article [1], it is reported that the image for Fig. 2D was inadvertently replaced with the image corresponding to Fig. 3D.

Fig. 2 has been corrected from:

**Fig. 2:**
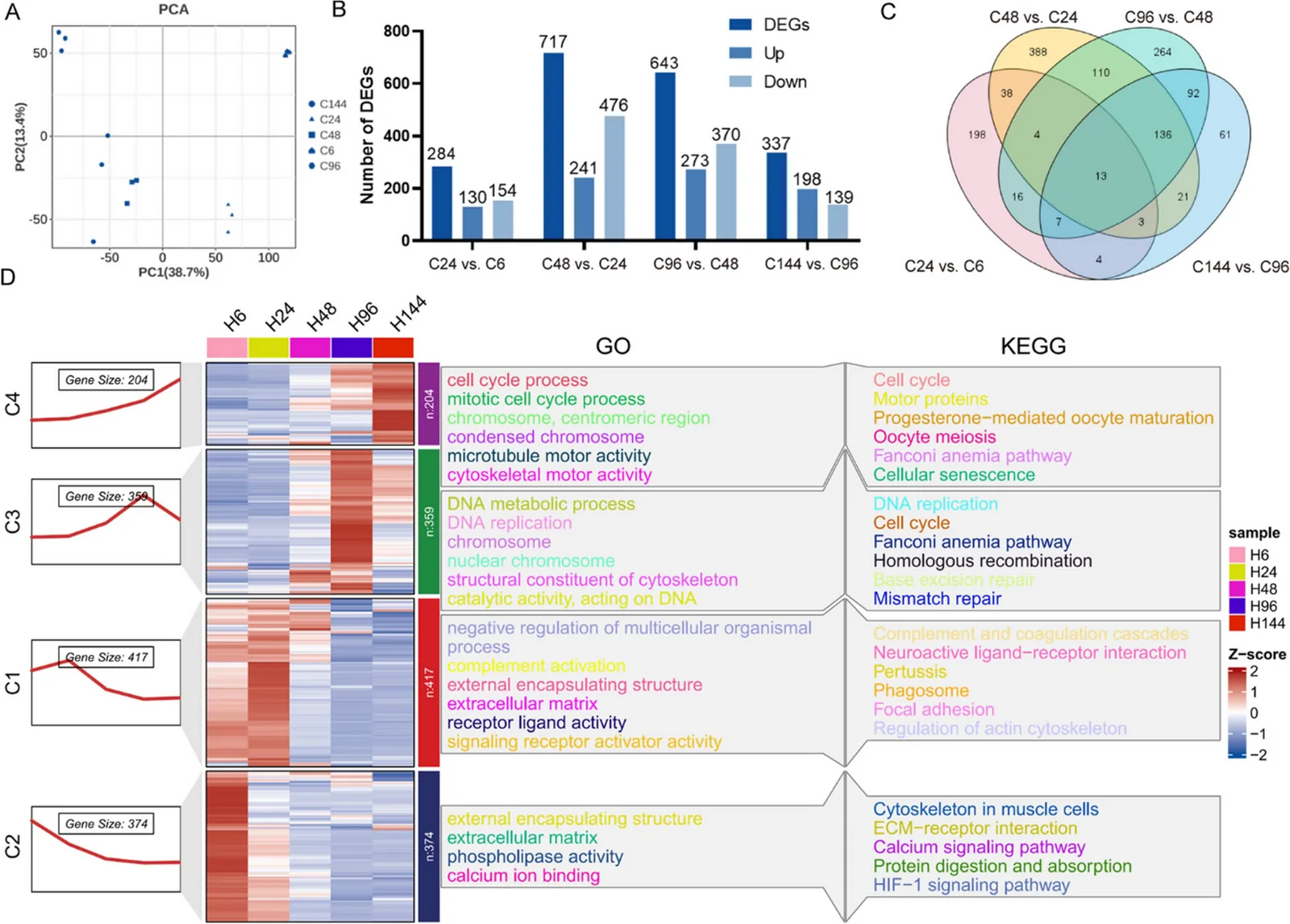
Identification, clustering, and functional enrichment analysis of DEGs at 37 °C for different culture durations. **A** PCA of samples in each group in transcriptomics. **B** The number of DEGs. **C** Venn diagram of DEGs. **D** Heatmap of the four clusters at five time points, and the top 5 significantly enriched GO pathways and KEGG pathways

To:

**Fig. 2:**
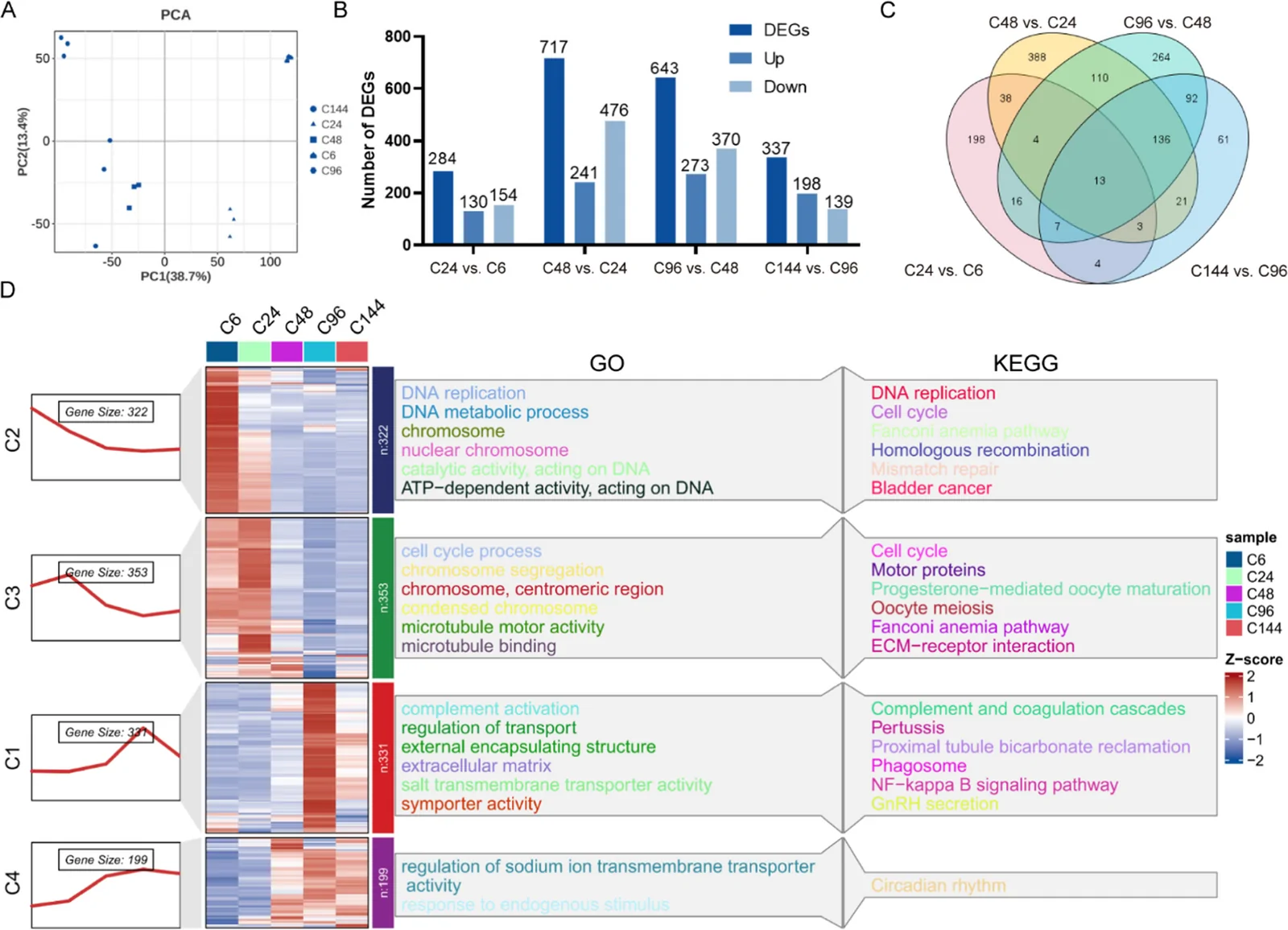
Identification, clustering, and functional enrichment analysis of DEGs at 37 °C for different culture durations. **A** PCA of samples in each group in transcriptomics. **B** The number of DEGs. **C** Venn diagram of DEGs. **D** Heatmap of the four clusters at five time points, and the top 5 significantly enriched GO pathways and KEGG pathways

Also, the imaging time points for the cell scratch assay in the Methods section has been corrected from “6, 24, 48, 96, and 144 h” to “0, 6, 12, 24, and 48 h”.

In Additional File 2, two authors were inadvertently omitted in title page, and Fig. S2 was inadvertently replaced with image corresponding to Fig. S1. The Additional File has been corrected.

The original article [1] has been corrected.

## References

[1] Yang X, Xie Y, Luo Y, et al. RFLNA mitigates heat stress-impaired chondrocyte proliferation and vertebral development through cytoskeletal regulation in pigs. J Animal Sci Biotechnol. 2026;17:68. 10.1186/s40104-026-01387-x. PMC13081642 41981703

