# Supplementary material for: RFLNA mitigates heat stress-impaired chondrocyte proliferation and vertebral development through cytoskeletal regulation in pigs
Source: J Anim Sci Biotechnol. 2026 Apr 15;17:68. doi: 10.1186/s40104-026-01387-x (PMC13081642; doi:10.1186/s40104-026-01387-x)
Supplement: Supplementary file 2 — Additional file 2: Fig. S1. Functional enrichment analyses of DEGs at distinct stages under 37 °C. Fig. S2. Functional enrichment analyses of DEGs at distinct stages under 41 °C culture. Fig. S3. Functional enrichment analyses of DEGs from 41 °C vs. 37 °C comparisons at corresponding time points. Fig. S4. Functional enrichment analyses of DEGs from 41 °C vs. 37 °C comparisons in the time-series clustering. Fig. S5. Detection of RFLNA overexpression and interference efficiency and expression of related marker genes. [file 40104_2026_1387_MOESM2_ESM.docx]

**RFLNA mitigates heat stress-impaired chondrocyte proliferation and vertebral development through cytoskeletal regulation in pigs**

Xiaoyang Yang ^1^, Yuxuan Xie ^1^, Yabiao Luo ^1^, Yubei Wang ^2^, Lixian Yang ^1^, Longmiao Zhang ^2^, Meiying Fang ^1,2*^

First Author: Xiaoyang Yang

*Corresponding Author: Meiying Fang

E-mail: meiying@cau.edu.cn

Tel: +86-010-62734943

Fax: +86-010-62734943

**Supplementary information**


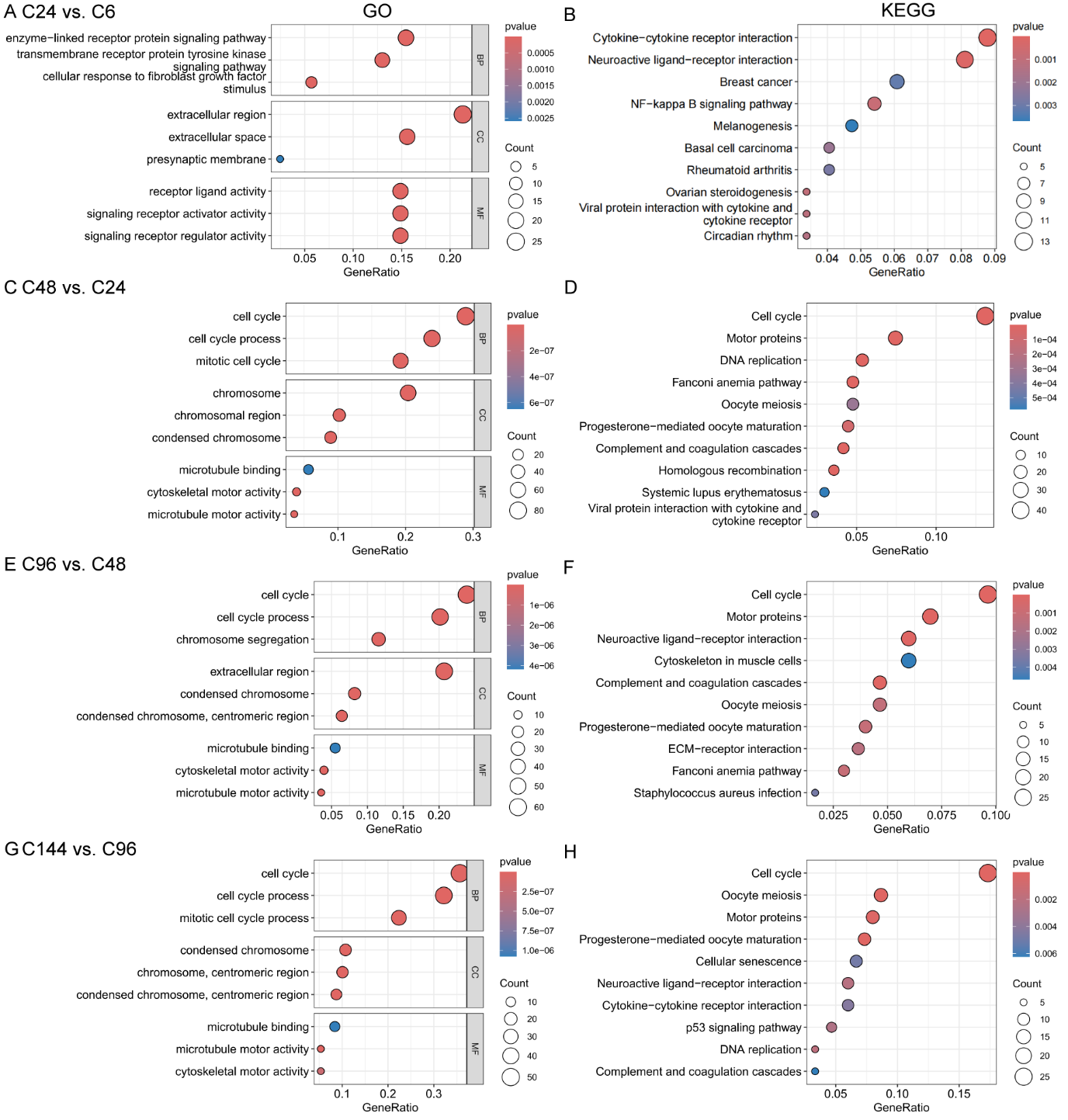


**Fig. S1** Functional enrichment analyses of DEGs at distinct stages under control-temperature. GO **A** and KEGG pathway enrichment **B** for DEGs in the C24 vs. C6 comparison group. GO **C** and KEGG **D** enrichment analyses for DEGs in the C48 vs. C24 group. GO **E** and KEGG **F** enrichment results for DEGs in the C96 vs. C48 group. GO **G** and KEGG **H** analyses for DEGs in the C144 vs. C96 group.


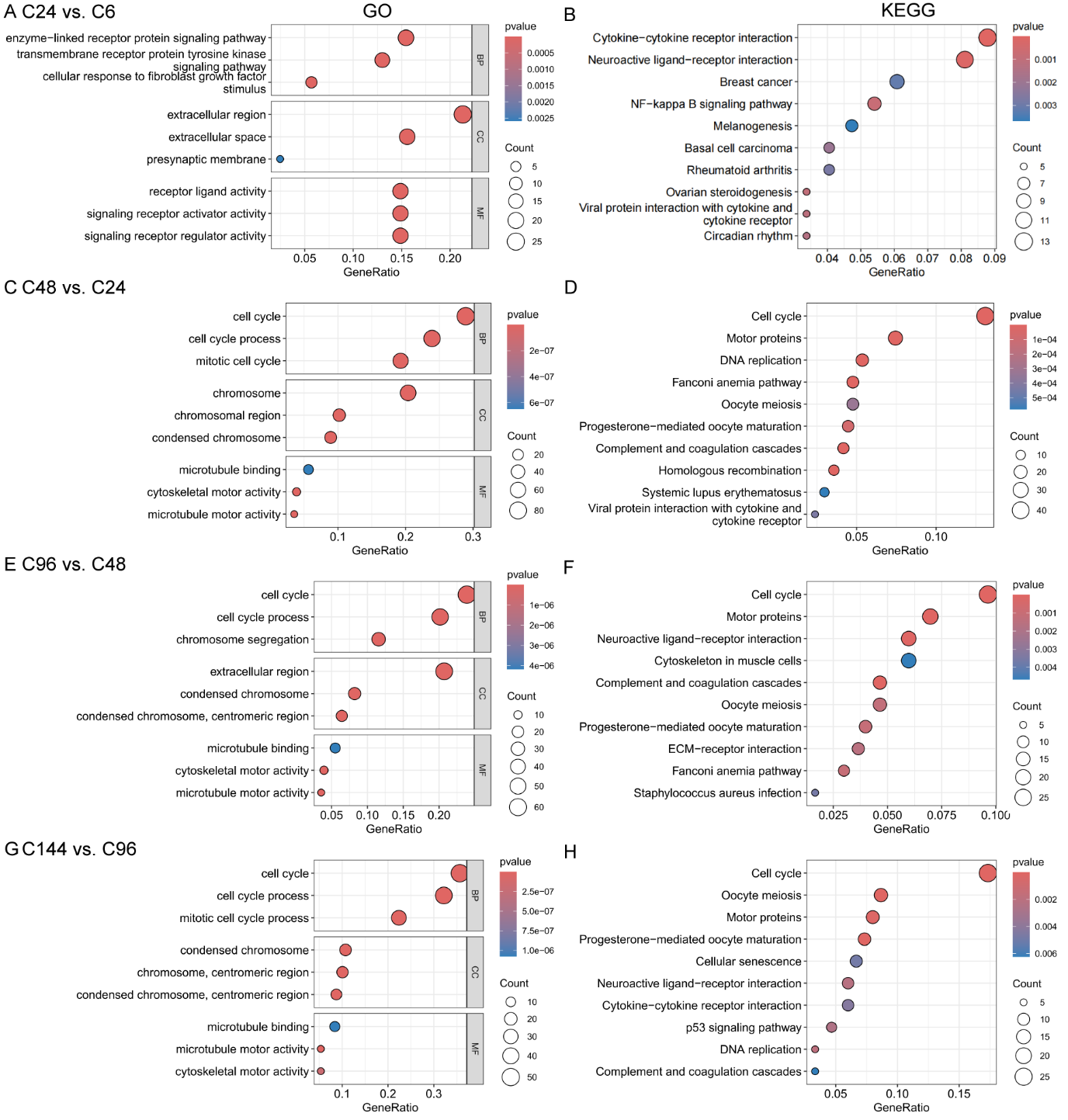


**Fig. S2** Functional enrichment analyses of DEGs at distinct stages under high-temperature culture. GO **A** and KEGG pathway enrichment **B** for DEGs in the H24 vs. H6 group. GO **C** and KEGG **D** enrichment results for DEGs in the H48 vs. H24 group. GO **E** and KEGG **F** analyses for DEGs in the H96 vs. H48 group. GO **G** and KEGG **H** enrichment profiles for DEGs in the H144 vs. H96 group.


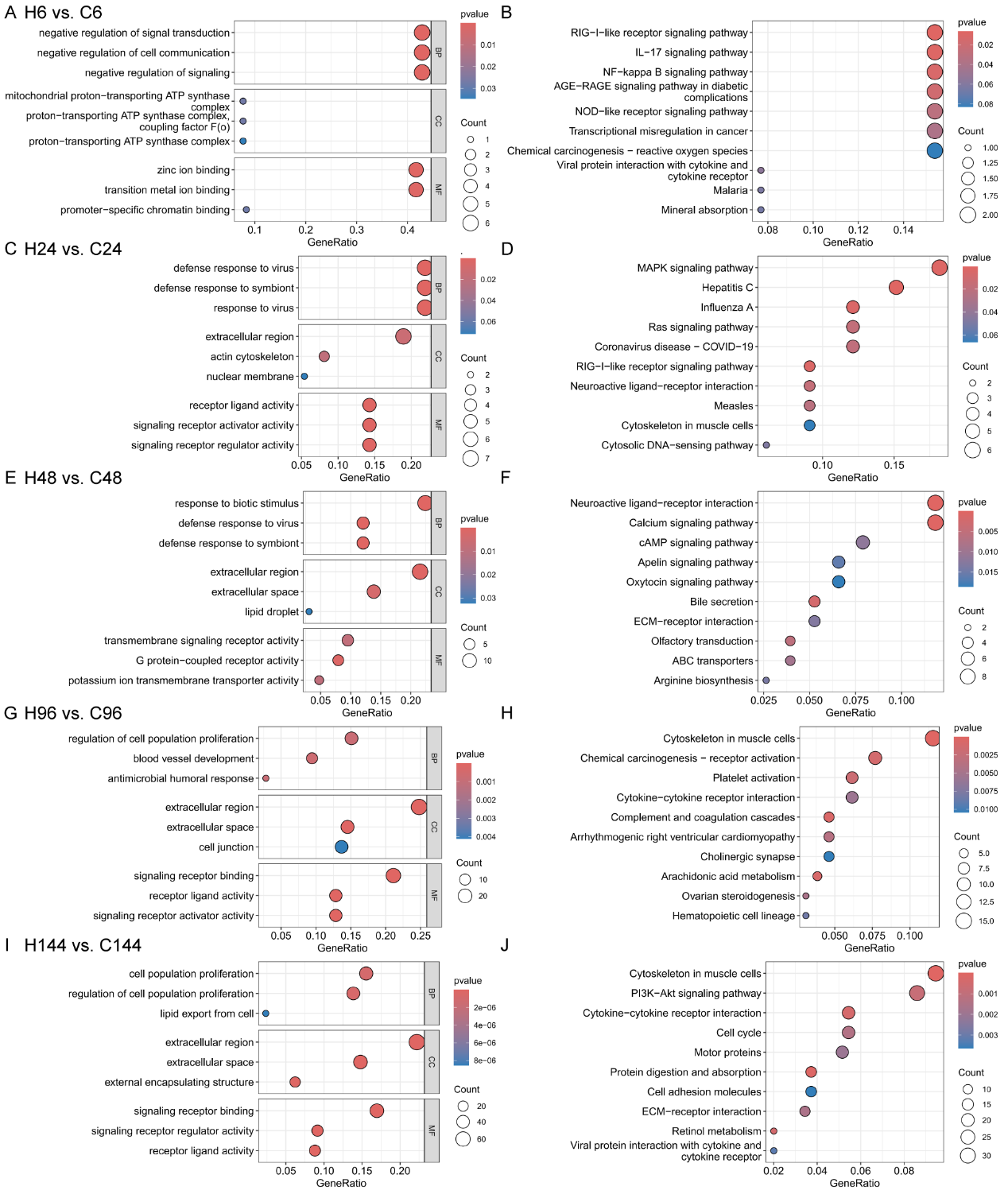


**Fig. S3** Functional enrichment analyses of DEGs from 41 °C vs. 37 °C comparisons at corresponding time points. GO **A** and KEGG pathway enrichment **B** for DEGs in the H6 vs. C6 group. GO **C** and KEGG **D** enrichment results for DEGs in the H24 vs. C24 group. GO **E** and KEGG **F** analyses for DEGs in the H48 vs. C48 group. GO **G** and KEGG **H** enrichment profiles for DEGs in the H96 vs. C96 group. GO **I** and KEGG **J** analyses for DEGs in the H144 vs. C144 group.


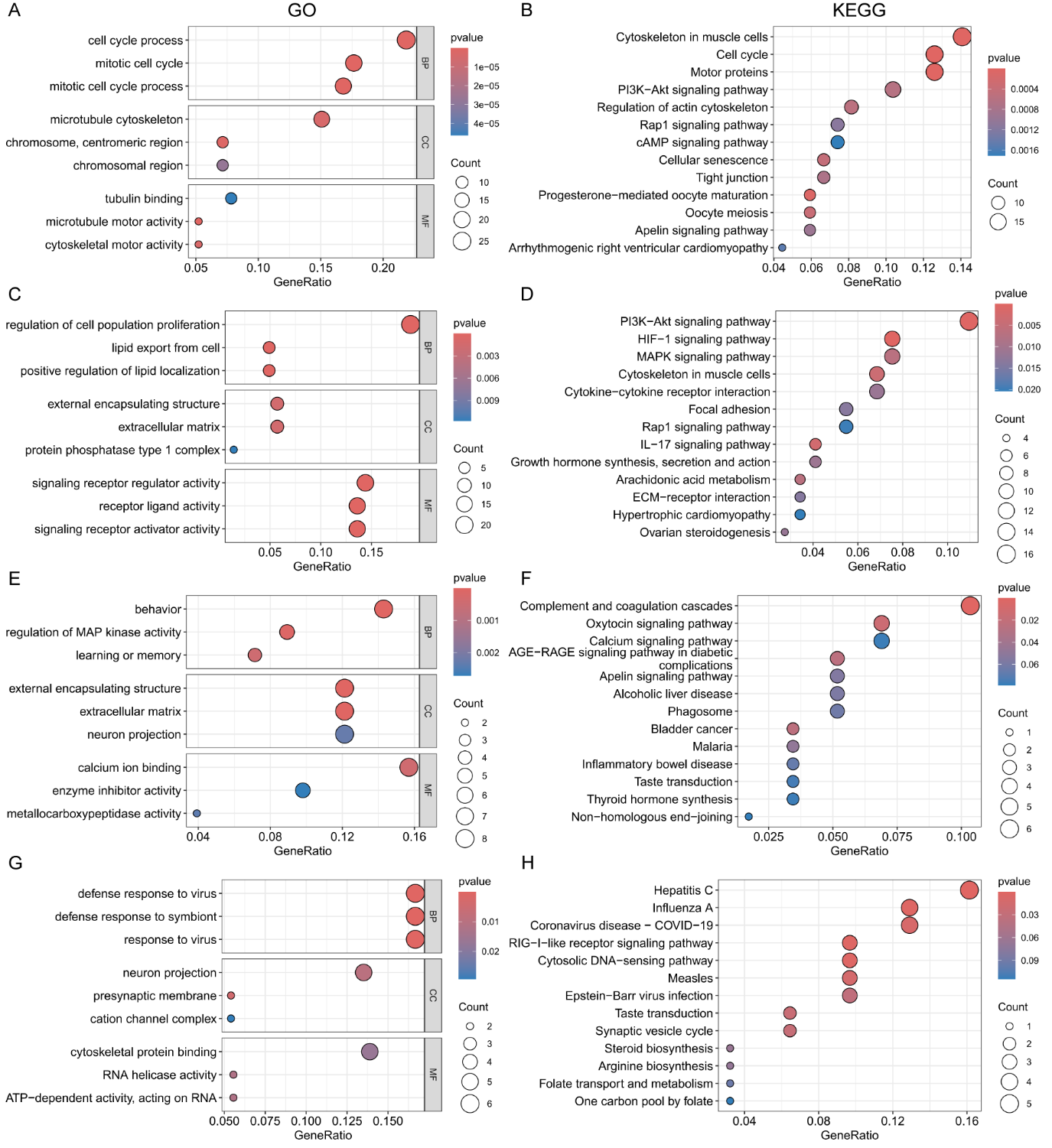


**Fig. S4** Functional enrichment analyses of DEGs from 41 °C vs. 37 °C comparisons in the time-series clustering. **A** GO enrichment analysis of the DEGs in Cluster 1 and **B** KEGG enrichment analysis. **C** GO enrichment analysis of the DEGs in Cluster 2 and **D** KEGG enrichment analysis. **E** GO enrichment analysis of the DEGs in Cluster 3 and **F** KEGG enrichment analysis. **G** GO enrichment analysis of the DEGs in Cluster 4 and **H** KEGG enrichment analysis.


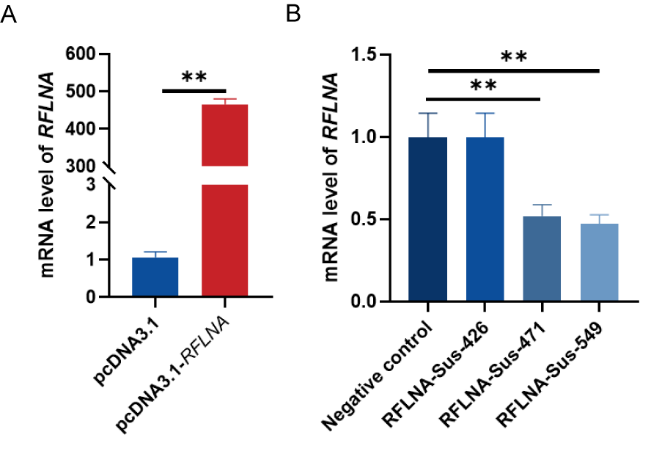


**Fig. S5** Detection of *RFLNA* overexpression and interference efficiency and expression of related marker genes. **A** Detection of *RFLNA* overexpression efficiency. **B** Detection of *RFLNA* interference efficiency.
