# Supplementary material for: RFLNA mitigates heat stress-impaired chondrocyte proliferation and vertebral development through cytoskeletal regulation in pigs
Source: J Anim Sci Biotechnol. 2026 Apr 15;17:68. doi: 10.1186/s40104-026-01387-x (PMC13081642; doi:10.1186/s40104-026-01387-x)
Supplement: Supplementary file 3 — Additional file 3. Semi-quantitative RT-PCR analysis of RFLNA expression in multiple tissues of 1-month-old Large White pigs, including heart, liver, spleen, lung, kidney, longissimus dorsi muscle, abdominal adipose, thoracic vertebra, lumbar vertebra, and articular cartilage. [file 40104_2026_1387_MOESM3_ESM.docx]

Semi-quantitative RT-PCR analysis of *RFLNA* expression in multiple tissues of 1-month-old Large White pigs, including heart, liver, spleen, lung, kidney, longissimus dorsi muscle, abdominal adipose, thoracic vertebra, lumbar vertebra, and articular cartilage.
